# CD49f and CD146: A Possible Crosstalk Modulates Adipogenic Differentiation Potential of Mesenchymal Stem Cells

**DOI:** 10.3390/cells13010055

**Published:** 2023-12-27

**Authors:** An Nguyen-Thuy Tran, Ha Yeong Kim, Se-Young Oh, Han Su Kim

**Affiliations:** 1 Department of Otorhinolaryngology-Head and Neck Surgery, College of Medicine, Ewha Womans University, Seoul 07985, Republic of Korea; antran@ewhain.net (A.N.-T.T.); ha0@ewha.ac.kr (H.Y.K.); 2 Graduate Program in System Health Science and Engineering, Ewha Womans University, Seoul 03760, Republic of Korea; 3 Department of Convergence Medicine, Ewha Womans University Mokdong Hospital, Ewha Womans University, Seoul 07985, Republic of Korea; ohs@ewha.ac.kr

**Keywords:** mesenchymal stem cell-specific markers, high-throughput screening, light scatter gating, CD49f, CD146

## Abstract

Background: The lack of appropriate mesenchymal stem cells (MSCs) selection methods has given the challenges for standardized harvesting, processing, and phenotyping procedures of MSCs. Genetic engineering coupled with high-throughput proteomic studies of MSC surface markers arises as a promising strategy to identify stem cell-specific markers. However, the technical limitations are the key factors making it less suitable to provide an appropriate starting material for the screening platform. A more accurate, easily accessible approach is required to solve the issues. Methods: This study established a high-throughput screening strategy with forward versus side scatter gating to identify the adipogenesis-associated markers of bone marrow-derived MSCs (BMSCs) and tonsil-derived MSCs (TMSCs). We classified the MSC-derived adipogenic differentiated cells into two clusters: lipid-rich cells as side scatter (SSC)-high population and lipid-poor cells as SSC-low population. By screening the expression of 242 cell surface proteins, we identified the surface markers which exclusively found in lipid-rich subpopulation as the specific markers for BMSCs and TMSCs. Results: High-throughput screening of the expression of 242 cell surface proteins indicated that CD49f and CD146 were specific for BMSCs and TMSCs. Subsequent immunostaining confirmed the consistent specific expression of CD49f and CD146 and in BMSCs and TMSCs. Enrichment of MSCs by CD49f and CD146 surface markers demonstrated that the simultaneous expression of CD49f and CD146 is required for adipogenesis and osteogenesis of mesenchymal stem cells. Furthermore, the fate decision of MSCs from different sources is regulated by distinct responses of cells to differentiation stimulations despite sharing a common CD49f^+^CD146^+^ immunophenotype. Conclusions: We established an accurate, robust, transgene-free method for screening adipogenesis associated cell surface proteins. This provided a valuable tool to investigate MSC-specific markers. Additionally, we showed a possible crosstalk between CD49f and CD146 modulates the adipogenesis of MSCs.

## 1. Introduction

Mesenchymal stem cells (MSCs) are multipotent adult stem cells located throughout vascularized tissues in the body [1,2]. They are one of the most widely used cell sources for cell-based therapy and regenerative medicine owing to their self-renewal, multi-potency, easily accessible and free of ethical issues [2,3,4]. Conventional methods for isolating MSCs generate a heterogeneous mixture of cells with various lineage commitments, reflected by differences in protein profiles, immunomodulatory capacities, and differentiation potentials [5,6,7], which might have a dramatic impact on the effectiveness of stem cell research and clinical applications. Numerous studies were performed to find a good approach to reduce inter-culture heterogeneity, including enrichment of a group of MSC subpopulations with specific features [8,9]. However, the lack of appropriate cell selection methods has given the challenges for standardized harvesting, processing, and phenotyping procedures of MSCs to gain greater clinical opportunities.

The cluster of differentiation (CD) antigen (also known as CD marker) is the most commonly used group of surface proteins to identify and investigate cell surface protein immunophenotypes. A classical set of CD markers has been committed as one of the minimal criteria for the identification of human MSCs, wherein MSCs must express CD105, CD73, CD90, and lack the expression of CD45, CD34, CD14 or CD11b, CD79α, or CD19, and HLA-DR surface molecules [10]. As of yet, the stemness-related surface markers remain to be found. Several groups used various antibody cocktails against cell surface markers to enrich a group of MSC subpopulations with higher differentiation potentials [11,12,13,14]. Alas, no single surface marker is capable of identifying cells that satisfy the minimal criteria of MSCs from various tissue sources.

In this regard, an increasing number of comparative studies have been conducted to analyze the similarities and differences of immunophenotype across stem cell populations, aiming to discover distinct surface markers of stem cells [15,16,17,18,19,20,21]. However, the adaptation of surface protein expression profiles depend on the surrounding environment and the mix of various lineage-committed cells in populations has hindered the success [22]. In order to provide a fairly homogeneous cell population for screening, some studies used genetic engineering to generate reporter cell lines that specifically mark a target cell type, followed by high-throughput technologies such as genomics, transcriptomics, and proteomics to discover unique surface markers for these special target cells. For example, clustered regularly interspaced short palindromic repeat (CRISPR) technology was performed to generate highly specified reporter cell lines that mark the chondroprogenitors [23], skeletal muscle progenitors [24], and dopamine progenitors [25]. This knock-in approach facilitated the screening of some lineage committed markers, although the technical limitations are the key factors making it less suitable to provide an appropriate starting material for a screening platform. Indeed, a more accurate, easily accessible approach is needed to solve these mentioned issues. 

Light scattering in flow cytometry analysis provides information about the size via forward scatter (FSC) and internal complexity or granularity of cells via side scatter (SSC). FSC versus SSC (FSC/SSC) gating is commonly used to discriminate subpopulations of blood cells. However, it is insufficient to identify subpopulations of MSCs owing to the lack of appreciable difference in size or internal complexity of cells regarding potentially different functionality across subpopulations. Recently, flow cytometry studies have described adipocytes as an SSC-high population [14,26,27]. Mesenchymal stem cells, as is well known, are one of the common precursors for adipocytes. Adipogenic-induced MSCs are characterized by intracellular accumulation of lipid droplets, which increases their internal complexity. Therefore, conducting a high-throughput, single-cell analysis of adipogenic-induced MSCs with FSC/SSC gating will facilitate the separation of differentiated MSCs. Taken this, we established a high-throughput screening approach with FSC/SSC gating to characterize the cell surface proteome of adipogenic differentiated MSCs. 

Bone marrow-derived MSCs (BMSCs) are one of the most well-studied types of MSCs. BMSCs are isolated from adult bone marrow aspirate through an invasive procedure. Meanwhile, tonsil-derived MSCs (TMSCs) are generally obtained from discarded tissue after tonsillectomy. Given that both MSCs are multipotent and have a perivascular origin, TMSCs are considered as an alternative source for MSC population owing to their easy accessibility and rapid self-renewal capacity [28]. In this study, we evaluated the surface protein profiles of BMSCs and TMSCs to provide a comparative and comprehensive characterization of MSCs from different tissue sources. 

## 2. Materials and Methods

### 2.1. Cell Culture

We used several lines obtained from different donors (2 BMSCs and 5 TMSCs). The donor information was provided in Supplementary Table S3. The BMSCs were purchased from PromoCell (Heidelberg, Germany). Tonsil-derived MSCs were thawed from a cell stock obtained from the patients using a study protocol approved by the Institutional Review Board (IRB) of Mokdong Hospital, Ewha Womans University (ECT 11-53-02) [29]. Written informed consent was obtained from all donors.

The TMSCs were cultured in Dulbecco’s modified Eagle’s medium containing high glucose (DMEM-HG; Welgene Inc., Gyeongsan, Republic of Korea) supplemented with 10% fetal bovine serum (FBS; Corning, NY, USA), 100 U/mL penicillin, 100 μg/mL streptomycin, and 0.25 μg/mL amphotericin B (Thermo Fisher Scientific, Waltham, MA, USA) at a density of 7 × 10^3^ cells per cm^2^. The BMSCs were maintained in Mesenchymal stem cell growth medium (PromoCell) supplemented with 100 U/mL penicillin, 100 μg/mL streptomycin, and 0.25 μg/mL amphotericin B at 37 °C in a humidified atmosphere containing 5% CO_2_. Cells were passaged when they reached 80% of confluence. The MSCs from passages 3, 6, and 7 were used for the experiments described herein.

### 2.2. Tri-Lineage Differentiation

The BMSCs and TMSCs were plated on a 6-well plate at a density of 100,000 cells per well. After 3 days, the cells were differentiated using StemPro^TM^ differentiation kits (Thermo Fisher Scientific) for 2 weeks. The differentiation potentials were analyzed by staining with Oil red O (adipogenesis) and Alizarin red S (osteogenesis). 

The BMSCs and TMSCs were chondrogenically differentiated in the standard pellet culture to detect proteoglycan. A total of 300,000 cells were centrifuged at 500× *g* for 10 min at room temperature to form a pellet. After 1 day, pellets were chondrogenically induced using the StemPro^TM^ differentiation kit for 3 weeks. Then, the pellets were sectioned and stained with Safranin-O to demonstrate the presence of proteoglycan.

The micromass culture model was used as previously described [30,31] to detect lineage marker protein expressions by western blotting. Briefly, BMSCs and TMSCs were resuspended in DMEM supplemented with 10% FBS, 100 U/mL penicillin, 100 μg/mL streptomycin, and 0.25 μg/mL amphotericin B at a concentration of 1 × 10^7^ cells/mL. Thirty microliter droplets of the cell suspension were spotted in a 6-well culture plate. The cells were adhered to culture dishes by incubating for 1 h at 37 °C in a humidified atmosphere containing 5% CO_2_, followed by the addition of 2 mL DMEM and culturing for another day. The cells were differentiated using StemPro^TM^ differentiation kits for 2 weeks. Then, cells were harvested and subjected to western blotting to detect lineage marker protein expressions. 

### 2.3. High-Throughput Screening of Cell Surface Marker Profile 

Flow cytometry analysis was performed to investigate the surface antigen profile of undifferentiated- and adipogenic-induced BMSCs or TMSCs. Briefly, 300,000 cells were stained with each antibody from the BD Lyoplate™ Human Cell Surface Marker Screening Panel (BD Biosciences, San Jose, CA, USA), a system consisting of 242 purified monoclonal antibodies and corresponding isotype controls, followed by an Alexa-647 conjugated secondary antibody. Non-specific fluorescence was determined using equal aliquots of unstained cell preparations. Data were obtained by analyzing 10,000 events on an ACEA NovoCyte 3000 flow cytometer (Agilent Technologies, Santa Clara, CA, USA). 

Cells were classified into lipid-rich cells as the SSC-high population and lipid-poor cells as the SSC-low population for adipogenic-induced BMSCs or TMSCs. Data were obtained and analyzed by an ACEA NovoCyte 3000 flow cytometer as described above. A comparative study of surface protein profile between undifferentiated and adipogenic-differentiated cells and between lipid-rich and lipid-poor cells was performed to identify the specific surface markers of MSCs. The proteins exclusively expressed by lipid-rich cells are considered adipogenesis-associated markers.

### 2.4. Immunocytochemistry

Cells were seeded on 12-well-plate at 50,000 cells per well until 70–80% confluency. Cells were induced for 2 weeks using a commercial StemPro^TM^ Adipogenesis Differentiation Kit. Then, cells were fixed with 4% paraformaldehyde and treated with 1% bovine serum albumin. Cells were incubated overnight with primary antibodies against surface antigens CD49f (BD Biosciences, #555734, 1:200) and CD146 (BD Biosciences, #550314, 1:200) at 4 °C, followed by an Alexa Fluor 594 conjugated secondary antibody (Thermo Fisher Scientific, #A11005, 1:200; #A11007, 1:200) or an Alexa Fluor 488 conjugated secondary antibody (Thermo Fisher Scientific, #A11006, 1:200) for 1 h at room temperature. Subsequently, 2 μM Bodipy 493/503 (Thermo Fisher Scientific) was used to label lipid droplets. The expression of surface antigens on stem cell-derived adipocytes was observed using an Eclipse Ti2-U inverted microscope (Nikon, Inc., Melville, NY, USA).

### 2.5. Fluorescence-Activated Cell Sorting (FACS) of MSCs

The BMSCs and TMSCs were obtained at passage 3. The cells were centrifuged, and the pellet was gently pipetted. Then, cells were stained with antibody cocktail against CD49f (BD Biosciences, #747725) and CD146 (BD Biosciences, #563619) at a concentration of 1 μg per 1 × 10^6^ cells on ice. Flow cytometric acquisition and cell sorting were performed using a BD FACSAria III Cell Sorter (BD Biosciences). The purity of sorted cell subsets was determined by post-sorting analysis. Sorted cells were expanded up to 3 passages to generate a sufficient number of cells for subsequent experiments. 

### 2.6. Western Blotting Analysis

Western blotting was performed using a standard protocol. Briefly, cells were washed twice with ice-cold phosphate buffered saline (2 g NaCl, 0.2 g KCl, 1.44 g Na_2_HPO_4_, and 0.24 g KH_2_PO_4_ in 1 L of deionized H_2_O at pH 7.5), lysed with lysis buffer (20 mM Tris-HCl at pH 7.5, 1% Triton X-100, 150 mM NaCl, 1 mM ethylenediaminetetraacetic acid, 1 mM ethylene glycol-bis(β-aminoethyl ether)-*N*,*N*,*N*′,*N*′-tetraacetic acid) supplemented with 5 protease inhibitor cocktails (cOmplete^TM^, EDTA-free protease inhibitor cocktail, Roche, #11873580001; 10 μM phenylmethanesulfonyl floride, Sigma-Aldrich, St. Louis, MO, USA, #P7626; 10 μM sodium fluoride, Sigma-Aldrich, #P7920; 10 μM sodium orthovanadate, Sigma-Aldrich, #S6508; and 10 μM glycerol-2-phosphate, Sigma-Aldrich, #G9891). The extracts were centrifuged at 12,000× *g* for 20 min at 4 °C. Total protein concentration was determined using the bicinchoninic acid (BCA) assay. The extracted proteins were resuspended in sodium dodecyl sulfate (SDS) sample buffer. Five to ten micrograms of proteins were resolved by SDS-polyacrylamide gel electrophoresis (SDS-PAGE) and transferred onto a 0.2 μm nitrocellulose membrane (Amersham, Piscataway, NJ, USA). The membrane was blocked with blocking solution (5% skim milk) for 1 h at room temperature, followed by incubation with primary antibody in blocking solution at 4 °C overnight. Membranes were washed 3 times with TBST (0.1% Tween 20/TBS buffer (2.4 g Tris and 8.8 g NaCl in 1 L of deionized H_2_O at pH 7.5)) and incubated with the horse radish peroxidase (HRP)-conjugated anti-mouse/rabbit secondary antibodies for 1 h at room temperature. The membrane was developed using Pierce ECL western blotting substrate (Thermo Fisher Scientific) and detected using a Chemidoc imaging system (Bio-Rad, Hercules, CA, USA). The following primary and secondary antibodies were used in this study: glyceraldehyde-3-phosphate dehydrogenase (GAPDH, Ab Frontier, #LF-PA0018, 1:3000), peroxisome proliferator-activated receptor γ (PPARγ, Cell Signaling, Danvers, MA, USA, #2435S, 1:1000), adiponectin (ADIPOQ, Cell Signaling, #2789T, 1:1000), fatty acid binding protein 4 (FABP4, Cell Signaling, #2120S, 1:1000), collagen type 1 (COL1A1, Cell Signaling, #72026S, 1:1000), osteocalcin (OCN, Abcam, #ab133612, 1:1000), integrin subunit alpha 6 (CD49f, Cell Signaling, #3750S, 1:1000), melanoma cell adhesion molecule (CD146, Cell Signaling, #13475, 1:1000), anti-mouse IgG HPR conjugated antibody (Bethyl Laboratories, #A90-116P, 1:3000), and anti-rabbit IgG HPR conjugated antibody (Bethyl Laboratories, #A120-101P, 1:3000). These antibodies were used for western blotting with human cells as validated in previous studies [32,33,34,35,36,37,38]. 

### 2.7. siRNA

siRNA specific for human CD49f, CD146, and non-targeting control siRNA were purchased from Santa Cruz Biotechnology (#sc-35918, #sc-43129, and #sc-37007). Transient transfection was performed by Lipofectamine^TM^ 2000 transfection reagent (Thermo Fisher Scientific) for 6 h. Cells were differentiated using StemPro^TM^ differentiation kits approximately 24 h post-transfection. Differentiation potentials of knockdown cells were analyzed by western blotting as described above.

### 2.8. Quantification and Statistical Analysis

Data are presented as the mean ± standard error of the mean (SEM). Statistical differences between groups were evaluated by one-way analysis of variance (ANOVA) with a Tukey-Kramer multiple comparisons test (* *p* < 0.05, ** *p* < 0.01, and *** *p* < 0.001). GraphPad Prism 9.0 statistical software (GraphPad Software, Inc., San Diego, CA, USA) was used for the analysis. A *p*-value < 0.05 was considered significant.

## 3. Results

### 3.1. Donor’s General Characteristics

**Figure 1:**
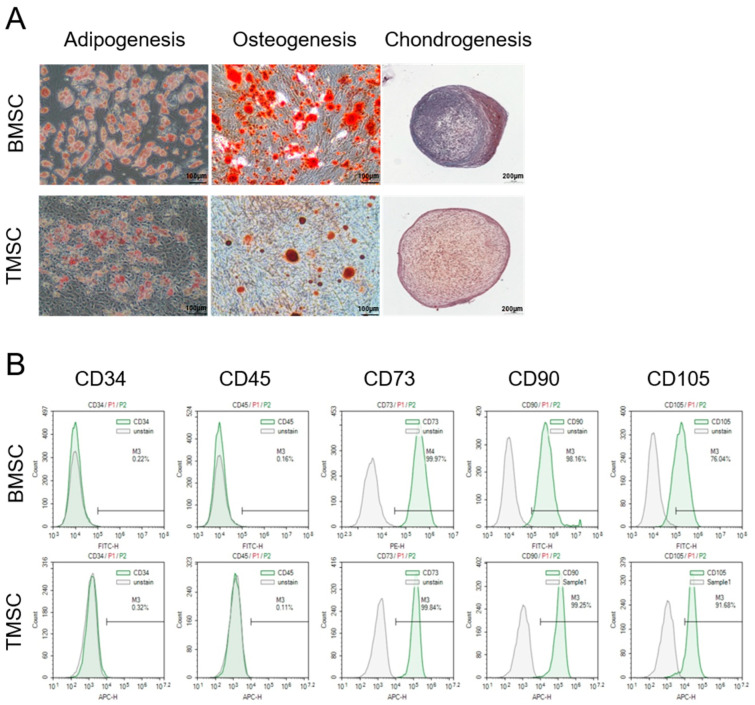
MSCs general characteristics. (**A**) Tri-lineage differentiation potential of BMSCs (upper panel) and TMSCs (lower panel) toward adipogenic, osteogenic and chondrogenic lineages was confirmed by staining with Oil red O (adipogenesis), Alizarin red S (osteogenesis), and Safranin O (chondrogenesis). Scale bar: adipogenesis and osteogenesis: 100 μm; chondrogenesis: 200 μm. (**B**) Flow cytometric analysis of BMSCs (upper panel) and TMSCs (lower panel). Both BMSCs and TMSCs expressed the classical set of mesenchymal stem cell surface markers, which is one of the minimal criteria for the identification of human MSCs proposed by the ISCT.

First, we examined the general characteristics to ensure that the cells meet the current minimal criteria for MSCs. Both BMSCs and TMSCs differentiated into adipogenic, osteogenic, and chondrogenic lineages (Figure 1A) and expressed the classical set of surface markers defining MSCs (Figure 1B). 

### 3.2. Cell Surface Proteome of BMSCs and TMSCs 

**Figure 2:**
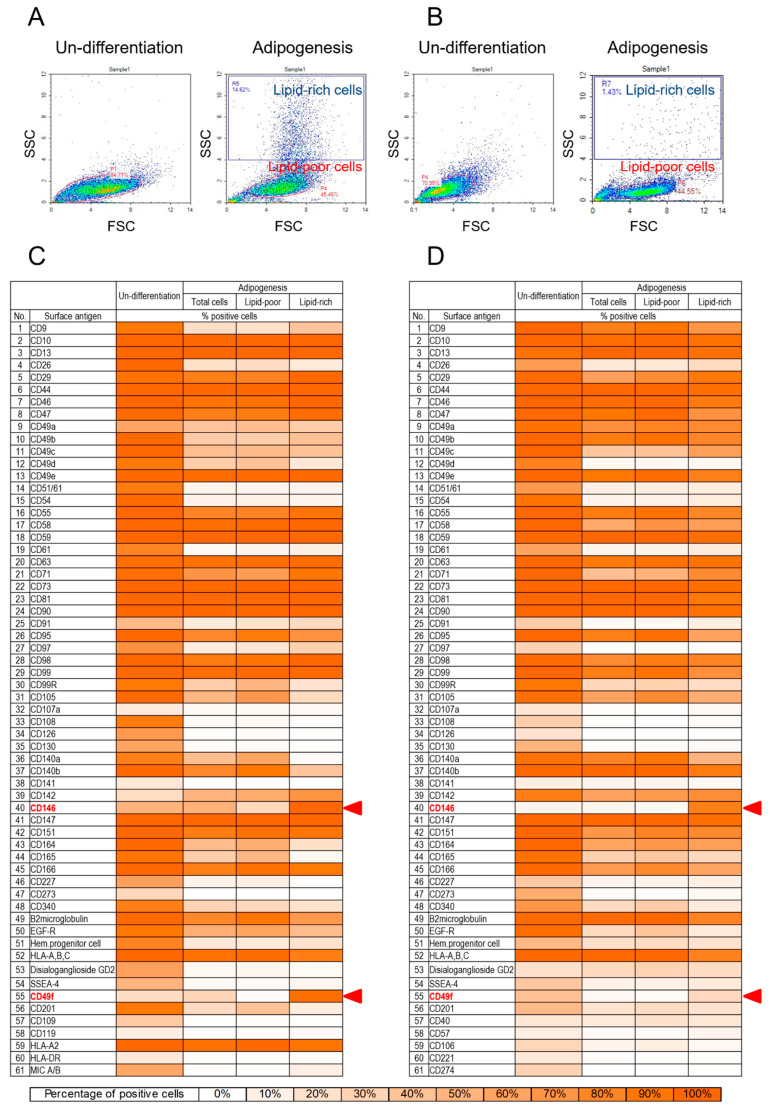
Generic method for FACS-based high-throughput screening of adipogenesis associated markers of MSCs. (**A**,**B**) Cell surface proteome of BMSCs (**A**) and TMSCs (**B**) were analyzed by flow cytometry. Adipogenic differentiated BMSCs and TMSCs were gated into lipid-rich cells as the SSC-high population and lipid-poor cells as the SSC-low population. The surface markers which exclusively found in lipid-rich subpopulation were considered as adipogenesis-associated markers. (**C**) Heatmap of the expression level of surface proteins on undifferentiated and adipogenic differentiated BMSCs. CD49f and CD146 were almost exclusively expressed in the lipid-rich population. The results are an average value from three independent experiments. (**D**) Heatmap of the expression level of surface proteins on undifferentiated and adipogenic differentiated TMSCs. CD49f and CD146 were almost exclusively expressed in the lipid-rich subpopulation. CD49f surface protein was significantly downregulated in adipogenic differentiated TMSCs. The results are an average value from three independent experiments.

High-throughput screening by flow cytometry was performed to examine the expression of 242 CD markers on MSCs and adipogenic-induced MSCs. We applied light scatter gating to classify the adipogenic induced cells into lipid-rich and lipid-poor subpopulations based on the differences in internal complexity of cells (Figure 2A,B). Lipid-rich cells were MSC-derived adipocytes characterized by intracellular accumulation of lipid droplets. Cells other than adipocytes were categorized into the lipid-poor subpopulation. Those cells served as the reference to identify specific markers that distinguish the differentiated cells from others. The surface marker expression profile of BMSCs was shown in the heatmap (Figure 2C). The entire dataset of BMSCs was listed in Supplementary Table S1. Undifferentiated BMSCs expressed 61 proteins (with over 10% positive cells) among the 242 cell surface proteins analyzed, including several previously reported cell surface markers [6,16,39,40]. We observed the tendency for downregulation of plasma membrane proteins upon differentiation, wherein CD9, CD26, CD49b, CD49c, CD49d, CD51/61, CD54, CD61, CD97, CD105, CD108, CD126, CD130, CD164, CD165, CD227, CD340, HPC, GD2, MIC A/B, and CD201 significantly lost their expression (over 50% of cells). In contrast, the expression levels of CD34, CD142, and CD271 increased by over 10% (Supplementary Table S1). Especially, CD49f and CD146 were predominantly expressed in the lipid-rich subpopulation (94.69 ± 3.01% and 99.38 ± 0.72%, respectively) and were weakly expressed in the lipid-poor subpopulation (3.12 ± 2.62% and 26.06 ± 3.67%, respectively) (Supplementary Table S1).

Tonsil-derived MSCs shared a similar cell surface proteome with BMSCs with 61 proteins expressed by over 10% of cells (Figure 2D, Supplementary Table S2). However, CD40, CD57, CD106, CD221, and CD274 were detected only in TMSCs (16.68 ± 6.1%, 11.25 ± 6.07%, 27.95 ± 9.69%, 15.19 ± 2.21%, and 29.9 ± 10.87%, respectively). In contrast, TMSCs lacked CD109, CD119, HLA-A2, HLA-DR, and MIC A/B, which were expressed by BMSCs (34.98 ± 18.17%, 11.36 ± 4.85%, 99 ± 0.72%, 15.32 ± 8.08%, and 58.49 ± 13.72%, respectively). CD49c, CD49d, CD51/61, CD54, CD61, CD71, CD99R, CD165, CD273, and EGF-R showed significantly reduced expression (over 50% of cells). Meanwhile, only CD39 showed an increase in expression level of over 10% (Supplementary Table S2). Importantly, CD49f and CD146 surface proteins were predominantly expressed in the lipid-rich subpopulation (25.7 ± 21.29% and 84.02 ± 11.25%, respectively), and their expression levels were minimal in the lipid-poor subpopulation (3.77 ± 4.76% and 4.34 ± 2.17%, respectively) (Supplementary Table S2), which was similar to that of BMSCs. However, the CD49f surface protein that was abundantly expressed on undifferentiated TMSCs (41.88 ± 5.35%) was significantly down-regulated on adipogenic-induced TMSCs (4.62 ± 5.35%) (Supplementary Table S2), unlike BMSCs. 

### 3.3. CD49f and CD146 Surface Proteins Exhibit a Distinct Expression Pattern on Adipogenic Differentiated MSCs 

**Figure 3:**
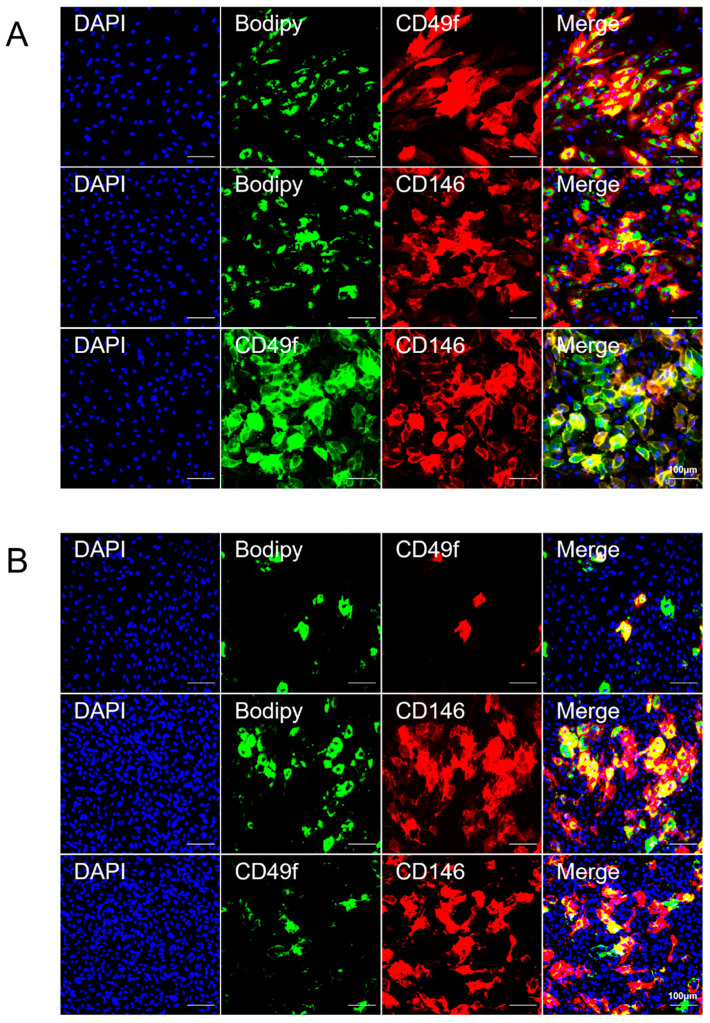
The distinct expression pattern of CD49f and CD146 on adipogenic differentiated MSCs. (**A**) Immunofluorescent imaging of adipogenic differentiated BMSCs. Cells were stained for DAPI (blue), Bodipy (green), and CD49f (red) or CD146 (red). Data show the colocalization of CD49f-stained (red) or CD146-stained (red) BMSCs with Bodipy (green). For double-staining, cells were stained for DAPI (blue), CD49f (green), and CD146 (red). Double-staining of CD49f (green) and CD146 (red) revealed that these two markers were almost colocalized. Scale bar: 100 μm. (**B**) Immunofluorescent imaging of adipogenic differentiated TMSCs. Cells were stained for DAPI (blue), Bodipy (green), and CD49f (red) or CD146 (red). Data show the colocalization of CD49f-stained (red) or CD146-stained (red) TMSCs with Bodipy (green). For double-staining, cells were stained for DAPI (blue), CD49f (green), and CD146 (red). The CD49f expression level was significantly downregulated. Scale bar: 100 μm.

Immunofluorescence staining was used to confirm the distinct expression pattern of identified surface proteins CD49f and CD146 on adipogenic differentiated BMSCs and TMSCs. Lipid droplets were labeled by Bodipy. CD49f and CD146 were clearly observed on lipid-rich cells by colocalization of these markers with Bodipy (Figure 3A,B). We did not detect CD49f or CD146 on lipid-poor cells (DAPI only). This was consistent with the data obtained from the surface protein screen. A similar pattern was observed in BMSCs and TMSCs isolated from various donors (Supplementary Figure S1). 

Double immunofluorescence staining of CD49f and CD146 revealed that these two markers were almost colocalized. Notably, simultaneous CD49f and CD146 double-stained cells were mainly observed in adipogenic-induced BMSCs (Figure 3A). Meanwhile, the loss of CD49f expression in TMSCs upon differentiation was shown by the absence of CD49f stained cells that were abundant in undifferentiated TMSCs. However, weak expression of CD49f was observed among CD146 stained cells (Figure 3B).

### 3.4. CD49f and CD146 Crosstalk Modulates Differentiation Potentials of MSCs

CD49f and CD146 play important roles in stem cell proliferation and differentiation [41,42,43,44,45]. However, crosstalk between these markers is unidentified. We sorted subpopulations from undifferentiated MSCs and examined their differentiation potentials upon adipogenesis and osteogenesis to investigate the possible roles of CD49f and CD146 in mesenchymal stem cell differentiation. 

**Figure 4:**
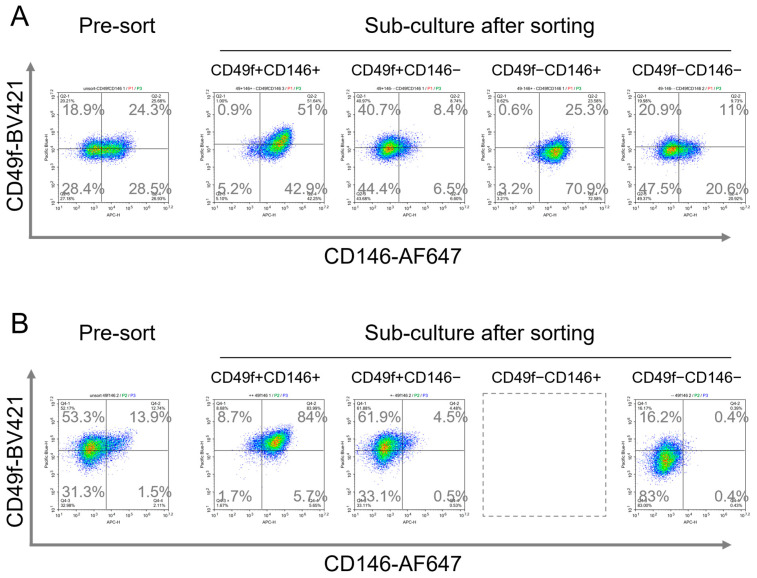
Flow cytometry immunophenotyping of the pre-sort and culture-expand subpopulations. (**A**) Pre-sort BMSCs consisted of CD49f^+^CD146^+^, CD49f^+^CD146^−^, CD49f^−^CD146^+^, and CD49f^−^CD146^−^ subpopulations. Sub-culture after sorting partially restored the expression levels of these markers. (**B**) Pre-sort TMSCs consisted of CD49f^+^CD146^+^, CD49f^+^CD146^−^, and CD49f^−^CD146^−^ subpopulations. Sub-culture after sorting partially restored the expression levels of these markers.

The immunophenotyping of pre-sorting BMSCs and TMSCs revealed that BMSCs consisted of CD49f^+^CD146^+^, CD49f^+^CD146^−^, CD49f^−^CD146^+^, and CD49f^−^CD146^−^ cells in the ratio of 24.3 ± 1.9%, 18.9 ± 1.9%, 28.5 ± 2.2%, and 28.4 ± 1.7% respectively (Figure 4A). Meanwhile, CD49f^+^CD146^−^ cells were the major subpopulation in TMSCs with 53.3 ± 1.1%, followed by CD49f^+^CD146^+^ and CD49f^−^CD146^−^ cells at 13.9 ± 1.1% and 31.3 ± 1.5% respectively, with no cells exhibited the CD49f^−^CD146^+^ immunophenotype (Figure 4B). A post-sort analysis showed that the subpopulation purity was greater than 97%. Sorted subpopulations were sub-cultured for 3 passages to obtain a sufficient number of cells for subsequent experiments. The sub-culturing partially restored the expression levels of these markers (Figure 4A,B).

**Figure 5:**
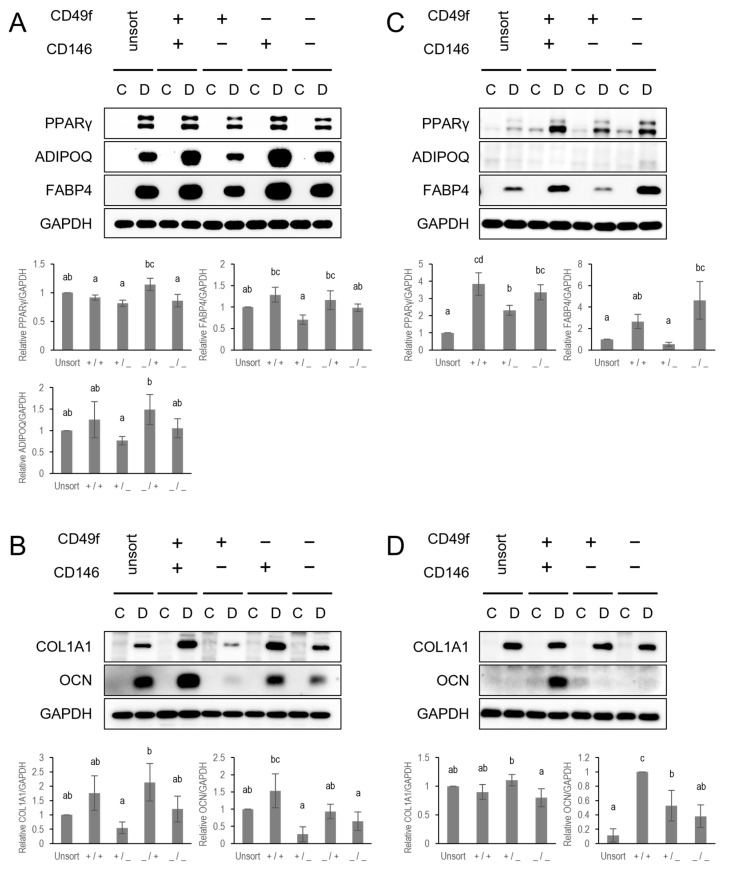
CD49f and CD146 crosstalk modulates differentiation potentials of MSCs. (**A**,**B**) Western blotting was used to assess the protein expression levels of adipogenic (**A**) and osteogenic (**B**) indicators in BMSCs after 14d of differentiation. C, control; D, differentiation. Bar charts show differentiation indicator quantitation normalized to GAPDH. The results are an average value from three independent experiments and presented as mean ± SD. Alphabet letters indicate statistically significant differences. Bars with different letters are considered statistically significant with *p* < 0.05. (**C**,**D**) Western blotting analysis was used to assess the protein expression levels of adipogenic (**C**) and osteogenic (**D**) indicators in TMSCs after 14 d of differentiation. C, control; D, differentiation. Bar charts show differentiation indicator quantitation normalized to GAPDH. The results are an average value from three independent experiments and presented as mean ± SD. Alphabet letters indicate statistically significant differences. Bars with different letters are considered statistically significant with *p* < 0.05. Full-length blots are presented in Supplementary Information: Full-length western blot images.

The adipogenic and osteogenic differentiation capabilities of subpopulations were elucidated by western blotting with lineage marker protein expressions (Figure 5). CD49f and CD146 identified subsets with varying differentiation potentials in BMSCs. CD146 enrichment enhanced adipogenesis and osteogenesis according to higher expression levels of adipogenic markers PPARγ, ADIPOQ, and FABP4 (Figure 5A), and osteogenic markers COL1A1 and OCN (Figure 5B) in CD146 enriched cells than in CD146 depleted cells. Meanwhile, the CD49f^+^CD146^−^ subpopulation showed low potentials in adipogenesis and osteogenesis (Figure 5A,B). In TMSCs, a significantly higher expression of adipogenic indicators were observed in CD49f^+^CD146^+^ cells than in CD49f^+^CD146^−^ cells. However, a similar amount of these markers was observed in CD49f^+^CD146^+^ and CD49f^−^CD146^−^ cells (Figure 5C). Validation by immunofluorescence staining, we observed many CD146^+^ cells within the CD49f^+^CD146^+^ sorted subpopulation did not co-localize with Bodipy (Supplementary Figure S2). This indicated that these cells were not adipocytes (white arrows). We also observed a low expression level of ADIPOQ in TMSCs (Figure 5C). For osteogenic potential, CD49f^+^CD146^+^ cells showed higher levels of OCN expression than other groups; however, COL1A1 expression levels did not significantly change among the groups (Figure 5D).

### 3.5. Knockdown of CD49f or CD146 Attenuates Adipogenesis and Osteogenesis Capabilities of BMSCs 

**Figure 6:**
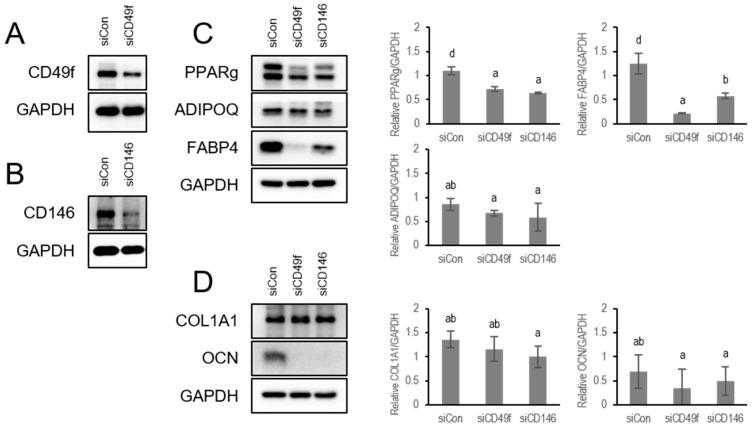
siRNA-mediated knockdown of CD49f or CD146 expression level in BMSCs. (**A**,**B**) Western blotting was used to analyze the protein expression levels of CD49f and CD146 after siRNA-mediated knockdown in BMSCs. (**C**,**D**) The effect of CD49f or CD146 knockdown on adipogenesis (**C**) and osteogenesis (**D**) of BMSCs. Bar charts show differentiation indicator quantitation normalized to GAPDH. The results are the average value from three independent experiments and presented as mean ± SD. Alphabet letters indicate statistically significant differences. Bars with different letters are considered statistically significant with *p* < 0.05. Full-length blots are presented in Supplementary Information: Full-length western blot images.

CD49f or CD146 expression levels of BMSCs were knocked down by transfecting them with siRNA against CD49f or CD146 to further elucidate whether CD49f and CD146 regulated the onset of mesenchymal stem cell differentiation. We observed a significant reduction of CD49f and CD146 in BMSCs (Figure 6A,B). Knockdown of either CD49f or CD146 attenuated adipogenesis (Figure 6C) and osteogenesis (Figure 6D) capabilities of BMSCs, as shown by lower expression levels of adipogenic markers PPARγ, ADIPOQ, and FABP4, and osteogenic markers COL1A1 and OCN in CD49f-siRNA (siCD49f) or CD146-siRNA (siCD146) transfected cells than in non-targeting control siRNA (siCon) transfected cells.

## 4. Discussion

We established a novel high-throughput screening strategy for identification of the adipogenesis-associated surface proteins, paving the way to elucidate the mesenchymal stem cell-specific markers. Typically, high-throughput screening of the surface protein profile of a pooled cell population provides very little information regarding potentially various identities across subpopulations of MSCs owing to stem cell heterogeneity. In this paper, adipogenic differentiated MSCs are separated by light scatter gating on account of the differences in internal complexity of cells, enables the comparative study of surface protein profiles between lipid-rich and lipid-poor cells. The primary advantage of this approach is that the need for reporter cell lines is eliminated. This improves the physiological relevance of the data and allows us to address many unanswered questions about mesenchymal stem cell immunophenotypes and identity.

A compilation of the results from the surface protein expression profile of lipid-rich and lipid-poor cells suggests that the surface markers CD49f and CD146, which are almost exclusively found in the lipid-rich subpopulation, are adipogenesis-associated markers. The expression of CD49f and CD146 on both undifferentiated and adipogenic induced cells facilitates sorting for the enrichment of MSCs. Subsequent investigations identified subsets with various differentiation potentials.

Indeed, understanding MSC surface protein profile holds great promise to predict their native physiological functions. In this study, both BMSCs and TMSCs show general properties of MSCs. Besides, BMSCs and TMSCs share similar cell surface protein repertoires with 56 common proteins following high-throughput screening of the panel consisting of 242 primary antibodies to surface proteins. Regarding the differences across MSCs, the lack of HLA-A2 and MIC A/B on TMSCs suggests different immunomodulatory properties between TMSCs and BMSCs [46,47]. Additionally, these cells exhibit discrepancies in the CD49f and CD146 immunophenotype during the undifferentiated state. Of note, BMSCs and TMSCs show different patterns of ADIPOQ and CD49f expression levels in response to adipogenic differentiation stimuli. Possibly, the tissue-specific peculiarities of MSCs may result in different immunophenotypes, secretome compositions, immunomodulatory properties and paracrine activities [48,49]. It is also possible that the age-related changes may result in variations in MSCs characteristics [50,51]. In the current study, BMSCs derived from old donors, TMSCs were isolated from young donors (Supplementary Table S3).

CD markers are the most commonly used group of surface proteins to characterize the cell surface proteome [17,18,52,53,54]. Among over 400 CD markers, CD146 [also known as melanoma cell adhesion molecule (MCAM)] stands out as the most promising identifier marker for human MSCs. CD146 is widely expressed on the surface of vascular endothelial cells [55], smooth muscle cells [56], pericytes [57], and MSCs isolated from various sources [58,59,60,61,62,63,64,65]. CD146^+^ MSCs show higher fibroblastic colony-forming unit frequency than the CD146^−^ group [66,67]. Notably, CD146^+^ clones from multiple tissue sources exhibit trilineage potency [68]. Loss of CD146 function impairs chondrogenic and myogenic differentiation in the mouse embryo cell line [69]. Most recently, it has been demonstrated that CD146 ablation suppresses adipogenesis, lipid accumulation, and enhances energy expenditure [70]. However, it is unclear whether CD146 alone is sufficient to identify MSCs. Previous studies showed that CD146^−^ and CD146^+^ MSCs exhibit similar tri-lineage differentiation potentials [67,71,72,73]. 

CD49f [also known as integrin α6 (ITGA6)] is a member of the integrin alpha chain family of proteins. As a matrix adhesion molecule, CD49f plays important roles in the proliferation and migration of stem cells [74]. Knockdown of CD49f results in the phosphorylation of focal adhesion kinase (FAK) and the reduction of NANOG, OCT4, and SOX2 in human pluripotent stem cells [44]. This suggests that CD49f plays roles in inactivating FAK signaling and supports stem cell self-renewal. Additionally, CD49f expression is sensitive to environmental changes [75]. Furthermore, the expression switch of CD49f shows dramatic consequences for cell proliferation/differentiation transition. In particular, CD49f increases during adipogenesis and treatment with CD49f-blocking antibody promotes pre-adipocytes to reenter the cell cycle [76]. However, these studies lack protein-protein interaction analysis; therefore, we cannot definitively conclude that CD49f solely regulates the cell fate decision. In fact, our results indicate that the CD49^+^CD146^−^ immunophenotype exhibits low adipogenesis and osteogenesis in BMSCs and TMSCs. 

As proposed by Chen et al. [77], adipogenic and osteogenic differentiation of MSCs is achieved by the actions of critical signaling pathways and key transcription factors. Therefore, we assume that CD49f and CD146 are among the mediators regulating the lineage commitment of MSCs via targeting key transcription factors such as PPARγ, C/EBPs, or RUNX2. In fact, the simultaneous expression of CD49f and CD146 is required for enhanced adipogenic and osteogenic differentiation shown in this study (Figure 5A) suggests a possible crosstalk between CD49f and CD146 surface markers. In addition, the switch-off of CD49f in CD49f^+^CD146^+^ enriched TMSCs results in loss of their adipogenic potential (Figure 5C), as shown by the maintenance of CD146^+^ MSCs in an undifferentiated state (Figure S2). This is consistent with previous study [76]. Taken together, we speculate that CD49f and CD146 act reciprocally to regulate adipogenic differentiation of MSCs. Furthermore, this study demonstrated that the differentiation potential of MSCs from different sources is regulated by distinct responses of cells to differentiation stimulations despite sharing a common CD49f^+^CD146^+^ immunophenotype. Further analysis is required to discover the underlying mechanisms regulating cell fate decision of MSCs isolated from diverse tissues. 

We could not investigate the change of CD49f and CD146 surface proteins before and after osteogenesis owing to limitations in single-cell dissociation of osteogenic differentiated MSCs. Nevertheless, our data reveal that CD49f^+^CD146^+^ enriched BMSCs and TMSCs exhibit higher osteogenic differentiation potential than other subgroups. This indicated that the CD49f^+^CD146^+^ immunophenotype may identify a high adipogenesis and osteogenesis subpopulation.

The knockdown of either CD49f or CD146 by transfection with siRNA attenuated adipogenesis and osteogenesis of BMSCs, although the decrease of ADIPOQ, COL1A1, and OCN expression levels were not statistically significant. A previous study shows that CD146 mRNA continuously increases from day 3 after adipogenic induction [70]. Therefore, we speculate that the transient effect of siRNA is not enough to maintain the significant decrease of adipogenesis and osteogenesis potentials in siCD49f or siCD146 transfected BMSCs. We suggest that a more stable genetic engineering system will help validate the role of CD49f and CD146 in regulating MSC differentiation.

## 5. Conclusions

We established an accurate, robust, transgene-free method for screening adipogenesis associated cell surface proteins. This provides a valuable tool to investigate MSC-specific markers. Additionally, we show that a possible crosstalk between CD49f and CD146 modulates the adipogenesis of MSCs. The simultaneous expression of CD49f and CD146 is required for adipogenesis and osteogenesis potentials. Further investigation is necessary to elucidate the mechanisms controlling MSC fate determination through CD49f and CD146 across MSCs from diverse tissues.

## Data Availability

The datasets used and/or analyzed during the current study are available from the corresponding author on reasonable request.

## Supplementary Materials

The following supporting information can be downloaded at: https://www.mdpi.com/article/10.3390/cells13010055/s1, Figure S1. The specificity of CD49f and CD146 on BMSCs (A) and TMSCs (B) isolated from various donors. Figure S2. The expression pattern of CD49f and CD146 on adipogenic-induced TMSC subpopulations. Immunofluorescent imaging of adipogenic differentiated TMSC subpopulations. Cells were stained with DAPI (blue), Bodipy (green), and CD49f or CD146 (red). Scale bar: 100 μm. We found many CD146+ cells within CD49f+CD146+ sorted subpopulation did not co-localize with Bodipy, indicating the non-adipocyte identity of these cells (white arrows). Table S1. The surface marker expression profile of BMSCs. Table S2. The surface marker expression profile of TMSCs. Table S3. Donor’s information.

## Abbreviations

BMSCs	bone marrow-derived mesenchymal stem cells
CD	the cluster of differentiation
CRISPR	clustered regularly interspaced short palindromic repeat
FAK	focal adhesion kinase
FSC/SSC	forward versus side scatter
FSC	forward scatter
ITGA6	integrin α6
MCAM	melanoma cell adhesion molecule
MSCs	mesenchymal stem cells
siCD146	CD146 small interfering RNA
siCD49f	CD49f small interfering RNA
siCon	non-targeting small interfering RNA
SSC	side scatter
TMSCs	tonsil-derived mesenchymal stem cells
