# Supplementary material for: CD49f and CD146: A Possible Crosstalk Modulates Adipogenic Differentiation Potential of Mesenchymal Stem Cells
Source: Cells. 2023 Dec 27;13(1):55. doi: 10.3390/cells13010055 (PMC10778538; doi:10.3390/cells13010055)
Supplement: Supplementary file 1 [file cells-13-00055-s001.zip › Supplemental information.pdf]

SUPPLEMENTAL INFORMATION

Supplemental Information includes two figures and three tables can be found with this article online.

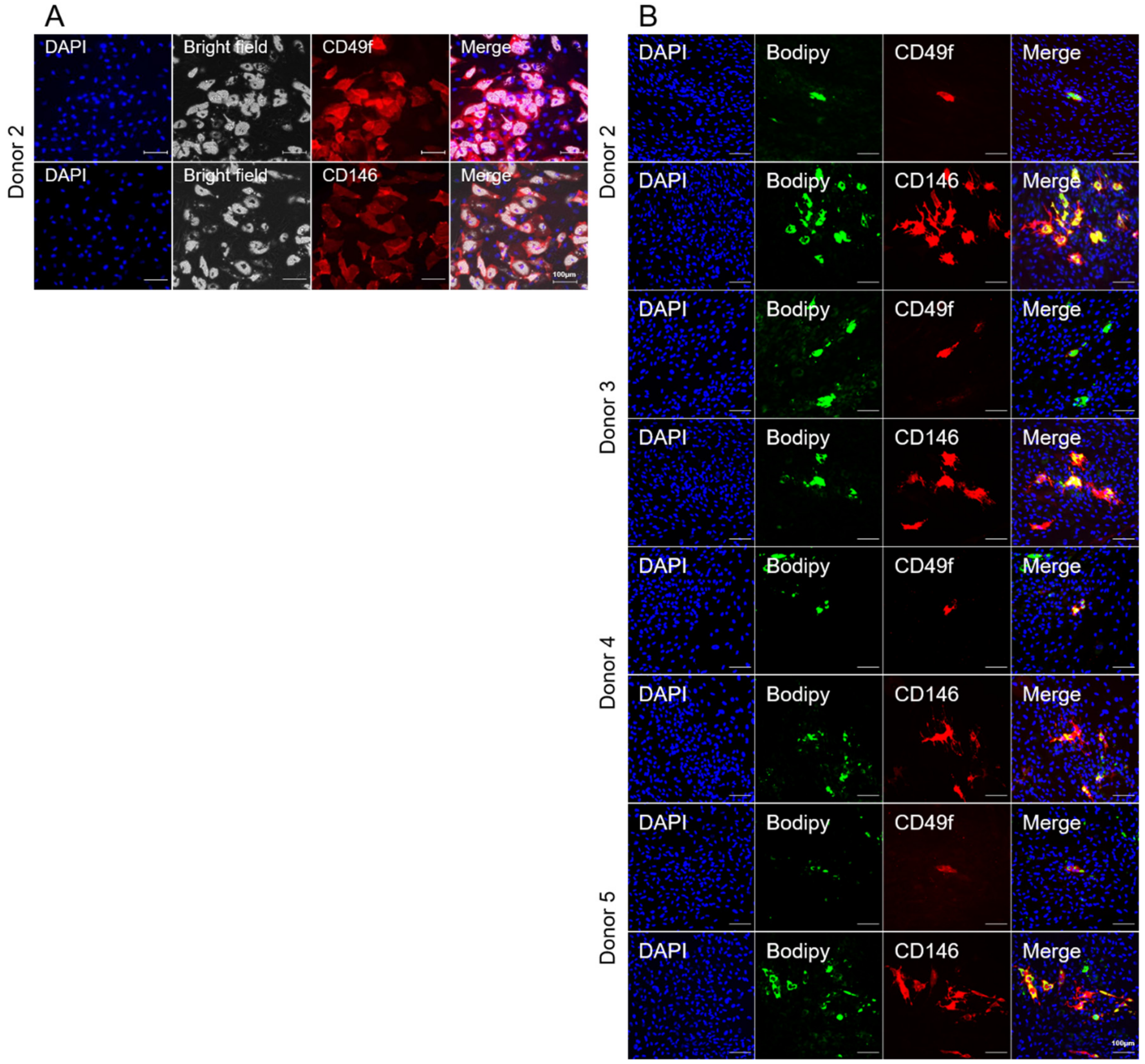

Figure S1. The specificity of CD49f and CD146 on BMSCs (A) and TMSCs (B) isolated from various donors. **A** Immunofluorescent imaging of adipogenic differentiated BMSCs. Cells were stained for DAPI (blue) and CD49f or CD146 (red). Lipid droplets appeared as highly refringent vacuoles within cytoplasm of BMSCs. Data show the colocalization of CD49f (red) or CD146 (red) with various adipogenic induced BMSCs. Donor 1 data were shown in Figure 3. Scale bar: 100μm. **B** Immunofluorescent imaging of adipogenic differentiated TMSCs. Cells were stained for DAPI (blue), Bodipy (green), and CD49f or CD146 (red). Data show the colocalization of CD49f (red) or CD146 (red) with Bodipy. Donor 1 data were shown in Figure 3. Scale bar: 100μm.

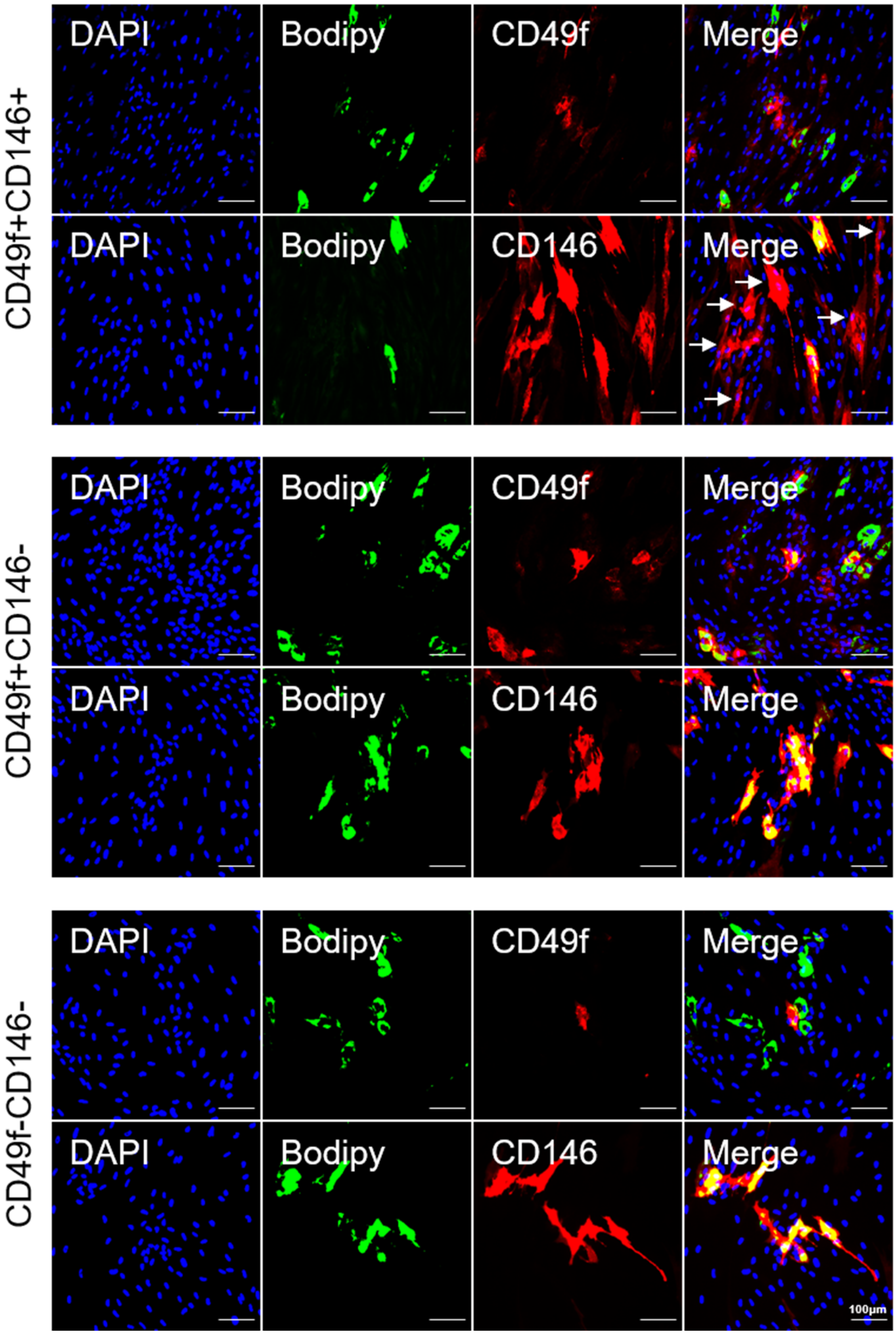

Figure S2. The expression pattern of CD49f and CD146 on adipogenic-induced TMSC subpopulations. Immunofluorescent imaging of adipogenic differentiated TMSC subpopulations. Cells were stained with DAPI (blue), Bodipy (green), and CD49f or CD146 (red). Scale bar: 100μm. We found many CD146+ cells within CD49f+CD146+ sorted subpopulation did not co-localize with Bodipy, indicating the non-adipocyte identity of these

cells (white arrows).

Table S1. The surface marker expression profile of BMSCs

		Un-differentiation		Adipogenesis					
				Total cells		Lipid-poor cells		Lipid-rich cells	
No.	Surface antigen	% positive	SD	% positive	SD	% positive	SD	% positive	SD
1	CD1a	0.19%	0.06%	0.18%	0.09%	0.11%	0.07%	0.12%	0.10%
2	CD1b	0.25%	0.02%	0.22%	0.09%	0.17%	0.10%	0.25%	0.09%
3	CD1d	0.24%	0.12%	0.15%	0.11%	0.12%	0.11%	0.20%	0.10%
4	CD2	0.13%	0.02%	0.18%	0.12%	0.12%	0.05%	0.23%	0.06%
5	CD3	0.23%	0.01%	0.20%	0.13%	0.18%	0.20%	0.42%	0.40%
6	CD4	0.14%	0.06%	0.18%	0.12%	0.16%	0.14%	0.21%	0.13%
7	CD4v4	0.70%	0.01%	0.32%	0.18%	0.34%	0.42%	0.34%	0.35%
8	CD5	0.14%	0.04%	0.12%	0.11%	0.11%	0.12%	0.18%	0.18%
9	CD6	0.15%	0.06%	0.13%	0.07%	0.09%	0.10%	0.24%	0.18%
10	CD7	0.21%	0.07%	0.15%	0.06%	0.14%	0.16%	0.13%	0.08%
11	CD8a	0.17%	0.04%	0.13%	0.04%	0.12%	0.09%	0.18%	0.11%
12	CD8b	0.28%	0.07%	0.19%	0.12%	0.15%	0.14%	0.10%	0.06%
13	CD9	88.46%	4.46%	22.49%	4.22%	20.89%	9.20%	38.67%	12.96%
14	CD10	99.55%	0.41%	97.66%	2.07%	98.77%	1.29%	98.77%	0.87%
15	CD11a	0.27%	0.05%	0.24%	0.09%	0.19%	0.18%	0.31%	0.04%
16	CD11b	0.42%	0.17%	0.12%	0.06%	0.09%	0.09%	0.08%	0.07%
17	CD11c	0.15%	0.05%	0.15%	0.06%	0.13%	0.12%	0.28%	0.23%
18	CD13	99.68%	0.39%	98.90%	1.52%	99.90%	0.09%	99.45%	0.69%
19	CD14	0.23%	0.06%	0.61%	0.67%	0.93%	1.48%	1.49%	2.14%
20	CD15	6.88%	1.53%	2.32%	1.20%	2.09%	2.20%	2.53%	1.59%
21	CD15s	0.16%	0.05%	0.29%	0.19%	0.29%	0.28%	0.17%	0.12%
22	CD16	0.09%	0.02%	0.16%	0.10%	0.14%	0.10%	0.30%	0.25%
23	CD18	0.10%	0.02%	0.17%	0.07%	0.18%	0.19%	0.35%	0.34%
24	CD19	0.15%	0.07%	0.18%	0.06%	0.16%	0.15%	0.18%	0.07%
25	CD20	0.47%	0.14%	0.21%	0.19%	0.22%	0.15%	0.17%	0.11%
26	CD21	0.15%	0.10%	0.16%	0.06%	0.10%	0.09%	0.10%	0.05%
27	CD22	0.15%	0.09%	0.16%	0.08%	0.18%	0.08%	0.20%	0.13%
28	CD23	0.10%	0.05%	0.14%	0.07%	0.12%	0.07%	0.26%	0.10%
29	CD24	0.24%	0.03%	0.22%	0.14%	0.16%	0.10%	0.23%	0.04%
30	CD25	0.13%	0.12%	0.16%	0.08%	0.16%	0.11%	0.23%	0.21%
31	CD26	98.21%	2.02%	21.05%	31.22%	23.83%	33.42%	14.39%	21.52%
32	CD27	0.75%	0.40%	0.41%	0.15%	0.35%	0.31%	0.58%	0.12%

33	CD28	0.18%	0.03%	0.12%	0.07%	0.21%	0.16%	0.34%	0.34%
34	CD29	93.30%	9.69%	84.34%	17.45%	81.83%	23.85%	98.51%	1.66%
35	CD30	0.13%	0.02%	0.13%	0.07%	0.12%	0.09%	0.10%	0.10%
36	CD31	0.52%	0.15%	0.19%	0.13%	0.17%	0.11%	0.19%	0.05%
37	CD32	0.17%	0.10%	0.20%	0.09%	0.16%	0.18%	0.16%	0.14%
38	CD33	0.22%	0.05%	0.20%	0.14%	0.15%	0.11%	0.14%	0.15%
39	CD34	4.69%	4.24%	16.26%	14.28%	16.32%	15.46%	14.65%	4.06%
40	CD35	0.17%	0.08%	0.26%	0.15%	0.66%	1.06%	2.35%	3.74%
41	CD36	9.25%	4.63%	17.86%	10.39%	12.60%	1.97%	38.99%	15.54%
42	CD37	0.12%	0.05%	0.17%	0.02%	0.15%	0.12%	0.22%	0.07%
43	CD38	0.94%	0.74%	0.53%	0.67%	0.42%	0.40%	0.50%	0.31%
44	CD39	0.21%	0.16%	0.87%	1.07%	1.13%	1.41%	0.20%	0.24%
45	CD40	2.40%	1.84%	0.30%	0.18%	0.24%	0.18%	0.26%	0.09%
46	CD41a	0.14%	0.02%	0.16%	0.06%	0.19%	0.28%	0.30%	0.15%
47	CD41b	0.13%	0.02%	0.17%	0.09%	0.14%	0.13%	0.14%	0.13%
48	CD42a	0.79%	0.01%	0.13%	0.09%	0.10%	0.09%	0.14%	0.14%
49	CD42b	0.14%	0.07%	0.19%	0.12%	0.14%	0.11%	0.25%	0.07%
50	CD43	0.09%	0.06%	0.22%	0.08%	0.21%	0.17%	0.22%	0.12%
51	CD44	99.51%	0.47%	99.20%	0.99%	99.87%	0.13%	99.37%	0.87%
52	CD45	0.18%	0.01%	2.00%	3.19%	3.60%	5.88%	5.31%	8.80%
53	CD45RA	0.26%	0.07%	0.16%	0.07%	0.18%	0.17%	0.30%	0.10%
54	CD45RB	0.11%	0.02%	0.17%	0.05%	0.12%	0.12%	0.16%	0.08%
55	CD45RO	0.84%	0.66%	0.30%	0.24%	0.30%	0.24%	0.43%	0.14%
56	CD46	99.51%	0.72%	91.58%	9.06%	93.32%	7.53%	99.40%	0.57%
57	CD47	97.66%	3.18%	84.18%	19.31%	86.03%	17.65%	95.97%	5.14%
58	CD48	0.33%	0.13%	0.39%	0.13%	0.45%	0.65%	1.25%	1.09%
59	CD49a	58.92%	26.93%	38.59%	17.90%	43.90%	16.06%	33.41%	15.25%
60	CD49b	98.29%	1.53%	34.64%	22.79%	33.09%	27.70%	42.24%	27.32%
61	CD49c	99.40%	0.49%	43.62%	26.91%	45.35%	28.03%	40.40%	21.89%
62	CD49d	88.52%	10.91%	33.54%	21.88%	40.59%	23.00%	16.04%	11.78%
63	CD49e	97.50%	2.97%	96.46%	4.59%	97.17%	2.99%	98.58%	0.88%
64	CD50	0.20%	0.03%	0.14%	0.11%	0.14%	0.13%	0.31%	0.31%
65	CD51/61	86.09%	9.14%	7.17%	8.43%	6.63%	6.04%	7.26%	2.75%
66	CD53	0.11%	0.02%	0.17%	0.07%	0.14%	0.14%	0.39%	0.30%
67	CD54	96.87%	3.25%	8.62%	7.07%	9.76%	8.70%	7.57%	1.19%
68	CD55	99.71%	0.28%	80.52%	14.03%	81.56%	14.91%	92.50%	4.01%
69	CD56	2.26%	1.04%	2.87%	4.46%	3.69%	5.99%	5.16%	8.52%
70	CD57	3.19%	1.78%	1.36%	0.49%	1.48%	0.51%	1.11%	0.34%

71	CD58	99.11%	1.30%	94.68%	7.15%	96.89%	3.73%	98.94%	1.39%
72	CD59	99.72%	0.41%	98.73%	1.53%	99.85%	0.14%	99.10%	0.28%
73	CD61	80.64%	15.37%	5.67%	7.70%	6.91%	11.13%	11.04%	16.71%
74	CD62E	0.14%	0.04%	0.23%	0.17%	0.15%	0.13%	0.21%	0.20%
75	CD62L	0.17%	0.06%	0.18%	0.08%	0.15%	0.12%	0.14%	0.12%
76	CD62P	0.19%	0.06%	0.17%	0.10%	0.12%	0.08%	0.27%	0.18%
77	CD63	94.80%	6.78%	92.87%	4.22%	94.78%	4.03%	96.36%	0.32%
78	CD64	0.17%	0.08%	0.12%	0.07%	0.14%	0.18%	0.21%	0.13%
79	CD66(a,c,d,e)	0.20%	0.07%	0.17%	0.04%	0.15%	0.10%	0.29%	0.17%
80	CD66b	0.43%	0.13%	0.14%	0.07%	0.16%	0.14%	0.43%	0.37%
81	CD66f	0.14%	0.02%	0.13%	0.03%	0.13%	0.15%	0.22%	0.18%
82	CD69	0.13%	0.06%	0.15%	0.02%	0.10%	0.12%	0.31%	0.12%
83	CD70	0.15%	0.03%	0.14%	0.10%	0.15%	0.17%	0.29%	0.20%
84	CD71	95.26%	5.42%	70.59%	9.27%	65.17%	20.44%	89.86%	9.45%
85	CD72	0.30%	0.07%	0.23%	0.18%	0.21%	0.19%	0.22%	0.12%
86	CD73	99.65%	0.24%	98.47%	1.51%	99.15%	0.72%	98.98%	0.15%
87	CD74	0.31%	0.16%	0.22%	0.05%	0.19%	0.04%	0.23%	0.07%
88	CD75	0.38%	0.16%	0.36%	0.23%	0.33%	0.23%	0.32%	0.18%
89	CD77	1.31%	0.19%	1.27%	0.21%	1.28%	1.36%	2.69%	1.85%
90	CD79b	2.14%	2.13%	1.09%	0.49%	0.75%	0.66%	1.92%	1.01%
91	CD80	0.09%	0.02%	0.11%	0.06%	0.08%	0.08%	0.18%	0.03%
92	CD81	99.19%	0.54%	98.02%	2.36%	98.27%	2.70%	98.85%	0.95%
93	CD83	0.58%	0.42%	0.13%	0.12%	0.25%	0.30%	1.18%	1.80%
94	CD84	0.38%	0.45%	0.20%	0.10%	0.28%	0.29%	0.61%	0.62%
95	CD85	0.18%	0.13%	0.13%	0.06%	0.14%	0.10%	0.12%	0.12%
96	CD86	0.14%	0.07%	0.12%	0.13%	0.11%	0.05%	0.08%	0.07%
97	CD87	0.11%	0.08%	0.11%	0.09%	0.09%	0.08%	0.12%	0.15%
98	CD88	0.22%	0.17%	0.12%	0.09%	0.14%	0.15%	0.22%	0.25%
99	CD89	0.19%	0.13%	0.18%	0.14%	0.18%	0.17%	0.22%	0.18%
100	CD90	99.72%	0.27%	98.60%	1.25%	99.90%	0.05%	99.42%	0.29%
101	CD91	46.29%	18.22%	14.23%	12.85%	19.34%	18.99%	24.49%	24.65%
102	CDw93	0.24%	0.16%	0.14%	0.08%	0.14%	0.14%	0.16%	0.19%
103	CD94	0.16%	0.16%	0.07%	0.09%	0.10%	0.09%	0.14%	0.13%
104	CD95	99.34%	0.71%	78.62%	12.07%	87.34%	15.40%	71.91%	5.63%
105	CD97	72.28%	9.39%	13.61%	13.69%	17.44%	11.69%	31.25%	32.77%
106	CD98	99.50%	0.55%	85.91%	11.35%	90.97%	6.56%	99.32%	0.28%
107	CD99	98.38%	2.16%	96.10%	3.41%	98.13%	0.27%	97.73%	1.63%
108	CD99R	88.07%	0.37%	33.72%	17.90%	48.82%	16.40%	18.77%	15.75%

109	CD100	0.15%	0.05%	0.13%	0.12%	0.15%	0.11%	0.07%	0.08%
110	CD102	9.82%	1.34%	0.26%	0.25%	0.20%	0.11%	0.10%	0.12%
111	CD103	0.10%	0.04%	0.17%	0.14%	0.16%	0.12%	0.17%	0.15%
112	CD105	97.98%	1.66%	48.53%	14.81%	67.98%	6.86%	24.49%	20.28%
113	CD106	6.57%	1.84%	0.92%	0.28%	1.81%	1.55%	0.33%	0.24%
114	CD107a	14.43%	8.71%	4.82%	2.73%	4.21%	0.42%	4.63%	2.10%
115	CD107b	5.57%	3.50%	3.40%	2.17%	1.98%	0.96%	3.40%	1.85%
116	CD108	87.53%	4.07%	3.47%	2.26%	5.82%	1.13%	1.80%	1.72%
117	CD109	34.98%	18.17%	0.44%	0.32%	0.83%	0.45%	0.56%	0.86%
118	CD112	0.92%	0.96%	0.15%	0.05%	0.21%	0.15%	0.12%	0.06%
119	CD114	0.15%	0.14%	0.11%	0.09%	0.15%	0.15%	0.25%	0.23%
120	CD116	0.20%	0.20%	0.10%	0.06%	0.12%	0.12%	0.19%	0.17%
121	CD117	0.11%	0.02%	0.12%	0.10%	0.09%	0.09%	0.10%	0.03%
122	CD118	0.15%	0.09%	0.15%	0.10%	0.14%	0.08%	0.09%	0.11%
123	CD119	11.36%	4.85%	1.71%	1.25%	2.19%	0.27%	3.51%	2.80%
124	CD120a	0.71%	0.06%	0.23%	0.10%	0.31%	0.34%	0.42%	0.49%
125	CD121a	5.03%	2.02%	0.88%	0.86%	1.29%	0.90%	0.10%	0.09%
126	CD121b	0.08%	0.08%	0.16%	0.16%	0.12%	0.10%	0.12%	0.11%
127	CD122	0.24%	0.23%	0.12%	0.08%	0.14%	0.15%	0.24%	0.21%
128	CD123	0.17%	0.13%	0.15%	0.11%	0.17%	0.10%	0.09%	0.11%
129	CD124	0.04%	0.00%	0.11%	0.09%	0.16%	0.14%	0.09%	0.10%
130	CD126	67.67%	3.85%	1.04%	0.87%	1.39%	0.55%	0.84%	0.90%
131	CD127	0.09%	0.06%	0.13%	0.07%	0.13%	0.11%	0.23%	0.07%
132	CD128b(CD182)	0.15%	0.05%	0.12%	0.12%	0.12%	0.14%	0.16%	0.26%
133	CD130	59.94%	33.52%	3.05%	2.87%	3.53%	2.25%	1.21%	1.77%
134	CD134	0.11%	0.08%	0.17%	0.10%	0.16%	0.21%	0.15%	0.02%
135	CD135	0.13%	0.09%	0.13%	0.09%	0.15%	0.13%	0.32%	0.25%
136	CD137	0.17%	0.09%	0.10%	0.01%	0.10%	0.13%	0.21%	0.08%
137	CD137 Ligand	0.10%	0.05%	0.14%	0.05%	0.19%	0.16%	0.15%	0.06%
138	CD138	0.11%	0.09%	0.35%	0.36%	0.43%	0.54%	0.25%	0.27%
139	CD140a	84.88%	13.79%	43.07%	22.99%	59.48%	6.19%	2.99%	1.77%
140	CD140b	99.00%	1.17%	79.10%	16.43%	89.65%	8.52%	38.76%	10.24%
141	CD141	16.74%	4.04%	0.66%	0.37%	1.28%	0.20%	0.48%	0.65%
142	CD142	23.43%	5.67%	49.90%	22.43%	55.71%	18.61%	71.15%	13.18%
143	CD144	0.22%	0.07%	0.15%	0.11%	0.16%	0.12%	0.13%	0.03%
144	CD146	50.22%	2.80%	52.25%	7.41%	26.06%	3.67%	99.38%	0.72%
145	CD147	99.70%	0.27%	98.69%	1.45%	99.88%	0.04%	99.47%	0.27%
146	CD150	0.40%	0.29%	0.15%	0.09%	0.12%	0.12%	0.16%	0.07%

147	CD151	98.35%	1.34%	86.16%	13.43%	93.63%	3.18%	91.49%	7.69%
148	CD152	0.44%	0.14%	0.15%	0.08%	0.18%	0.19%	0.42%	0.43%
149	CD153	0.43%	0.23%	1.47%	0.76%	1.10%	1.66%	3.29%	2.64%
150	CD154	0.14%	0.10%	0.10%	0.05%	0.11%	0.07%	0.10%	0.03%
151	CD158a	0.11%	0.02%	0.14%	0.09%	0.20%	0.13%	0.19%	0.15%
152	CD158b	0.14%	0.07%	0.11%	0.10%	0.10%	0.09%	0.16%	0.10%
153	CD161	0.07%	0.03%	0.08%	0.04%	0.20%	0.25%	0.62%	0.91%
154	CD162	7.30%	0.72%	0.33%	0.21%	0.15%	0.17%	1.07%	0.45%
155	CD163	0.11%	0.06%	0.17%	0.12%	0.18%	0.20%	0.15%	0.08%
156	CD164	97.69%	2.62%	44.67%	19.17%	54.97%	8.61%	20.95%	10.87%
157	CD165	91.29%	8.79%	32.25%	22.60%	47.71%	15.61%	4.74%	2.91%
158	CD166	99.07%	0.99%	84.04%	19.53%	93.60%	4.53%	88.85%	13.41%
159	CD171	0.10%	0.03%	0.17%	0.14%	0.18%	0.16%	0.12%	0.18%
160	CD172b	0.07%	0.02%	0.15%	0.10%	0.14%	0.12%	0.30%	0.18%
161	CD177	0.10%	0.05%	0.10%	0.11%	0.11%	0.08%	0.05%	0.06%
162	CD178	0.09%	0.02%	0.12%	0.09%	0.13%	0.11%	0.37%	0.34%
163	CD180	0.09%	0.05%	0.13%	0.08%	0.16%	0.14%	0.17%	0.15%
164	CD181	1.49%	1.75%	0.36%	0.34%	0.72%	0.95%	1.26%	2.03%
165	CD183	1.01%	0.30%	0.85%	0.53%	1.27%	1.69%	2.27%	3.22%
166	CD184	0.18%	0.16%	0.18%	0.10%	0.19%	0.23%	0.38%	0.37%
167	CD193	0.47%	0.46%	0.18%	0.18%	0.20%	0.13%	0.27%	0.17%
168	CD195	0.56%	0.71%	0.15%	0.11%	0.13%	0.17%	0.08%	0.09%
169	CD196	0.28%	0.03%	0.17%	0.06%	0.13%	0.11%	0.24%	0.06%
170	CD197	0.28%	0.25%	0.29%	0.24%	0.28%	0.32%	0.20%	0.14%
171	CD200	0.66%	0.23%	0.24%	0.26%	0.31%	0.29%	0.09%	0.16%
172	CD205	6.79%	4.52%	0.43%	0.43%	1.16%	0.93%	0.15%	0.11%
173	CD206	0.09%	0.01%	0.16%	0.11%	0.09%	0.11%	0.11%	0.12%
174	CD209	2.84%	3.45%	2.88%	1.98%	1.84%	1.86%	4.20%	2.87%
175	CD220	0.14%	0.05%	0.17%	0.12%	0.17%	0.19%	0.35%	0.36%
176	CD221	0.34%	0.13%	0.11%	0.11%	0.11%	0.10%	0.14%	0.07%
177	CD226	0.08%	0.04%	0.17%	0.09%	0.17%	0.13%	0.33%	0.16%
178	CD227	62.45%	30.12%	8.27%	8.52%	10.76%	12.13%	0.82%	0.48%
179	CD229	0.12%	0.10%	0.13%	0.08%	0.15%	0.17%	0.22%	0.20%
180	CD231	0.08%	0.01%	0.13%	0.09%	0.14%	0.12%	0.12%	0.14%
181	CD235a	0.23%	0.20%	0.23%	0.18%	0.26%	0.26%	0.15%	0.08%
182	CD243	0.27%	0.29%	0.15%	0.09%	0.22%	0.24%	0.25%	0.25%
183	CD244	0.12%	0.08%	0.14%	0.09%	0.18%	0.15%	0.34%	0.34%
184	CD255	0.13%	0.06%	0.18%	0.07%	0.17%	0.19%	0.19%	0.09%

185	CD268	0.08%	0.02%	0.13%	0.12%	0.15%	0.13%	0.17%	0.16%
186	CD271	1.06%	0.44%	14.81%	8.56%	17.10%	7.90%	5.22%	2.12%
187	CD273	26.62%	18.09%	0.15%	0.08%	0.17%	0.20%	0.22%	0.16%
188	CD274	1.26%	1.05%	0.12%	0.10%	0.14%	0.17%	0.08%	0.07%
189	CD275	1.81%	2.78%	0.16%	0.10%	0.18%	0.17%	0.15%	0.11%
190	CD278	0.10%	0.04%	0.13%	0.05%	0.09%	0.12%	0.26%	0.04%
191	CD279	0.16%	0.05%	0.21%	0.19%	0.13%	0.12%	1.00%	1.71%
192	CD282	0.18%	0.05%	0.17%	0.06%	0.13%	0.05%	0.63%	0.90%
193	CD305	0.23%	0.02%	0.40%	0.17%	0.42%	0.62%	3.60%	5.09%
194	CD309	0.26%	0.15%	0.20%	0.12%	0.16%	0.09%	0.33%	0.53%
195	CD314	0.17%	0.06%	0.30%	0.30%	0.20%	0.31%	1.44%	2.23%
196	CD321	3.19%	1.29%	8.40%	5.15%	6.82%	9.78%	37.37%	37.50%
197	CDw327	0.18%	0.02%	0.24%	0.13%	0.32%	0.32%	1.30%	2.12%
198	CDw328	0.54%	0.31%	0.31%	0.30%	0.17%	0.20%	1.24%	1.98%
199	CD329	0.39%	0.37%	0.18%	0.20%	0.17%	0.23%	0.42%	0.68%
200	CD335	0.29%	0.17%	0.22%	0.18%	0.18%	0.21%	1.05%	1.74%
201	CD336	0.29%	0.21%	0.11%	0.11%	0.05%	0.05%	0.10%	0.09%
202	CD337	0.36%	0.06%	2.42%	0.94%	1.51%	2.14%	2.08%	1.71%
203	CD338	0.29%	0.04%	0.42%	0.53%	0.40%	0.50%	2.72%	4.59%
204	CD340	84.36%	8.93%	29.16%	48.64%	26.18%	28.49%	20.07%	33.89%
205	abTCR	1.09%	1.43%	0.31%	0.26%	0.28%	0.42%	1.10%	1.70%
206	b2microglobulin	97.81%	1.88%	78.85%	18.65%	91.02%	2.84%	67.59%	30.62%
207	BLTR-1	0.21%	0.07%	0.21%	0.21%	0.17%	0.21%	0.86%	1.40%
208	CLIP	0.37%	0.14%	0.48%	0.62%	0.31%	0.43%	0.36%	0.49%
209	CMRF-44	0.19%	0.04%	0.24%	0.21%	0.26%	0.36%	1.29%	2.08%
210	CDRF-56	1.23%	1.50%	0.27%	0.18%	0.30%	0.26%	1.13%	1.61%
211	EGF-R	88.97%	12.30%	52.95%	34.18%	60.49%	13.88%	44.53%	41.50%
212	fMLP-R	0.51%	0.56%	0.18%	0.17%	0.21%	0.20%	0.56%	0.90%
213	gdTCR	0.37%	0.46%	0.05%	0.06%	0.01%	0.02%	0.04%	0.04%
214	Hem.progenitor cell	81.62%	4.91%	15.77%	18.62%	15.01%	12.06%	19.50%	27.72%
215	HLA-A,B,C	99.26%	0.59%	92.89%	5.76%	95.90%	3.06%	84.83%	19.90%
216	HLA-A2	99.00%	0.72%	93.82%	5.41%	98.11%	0.89%	91.26%	11.37%
217	HLA-DQ	0.28%	0.14%	0.42%	0.45%	0.45%	0.59%	2.37%	3.92%
218	HLA-DR	15.32%	8.08%	1.01%	0.88%	0.91%	0.41%	2.08%	3.20%
219	HLA-DR,DP,DQ	5.80%	3.55%	0.68%	0.58%	0.62%	0.40%	2.69%	4.26%
220	Invariant NKT	2.02%	3.04%	0.19%	0.11%	0.14%	0.22%	0.46%	0.58%

221	Disialoganglioside GD2	62.36%	9.03%	5.54%	3.16%	6.32%	0.54%	3.65%	3.38%
222	MIC A/B	58.49%	13.72%	1.55%	2.42%	1.19%	1.90%	10.97%	18.71%
223	NKB1	0.21%	0.17%	0.14%	0.06%	0.16%	0.20%	0.65%	0.96%
224	SSEA-1	8.42%	3.60%	3.75%	4.60%	2.96%	4.15%	5.52%	8.01%
225	SSEA-4	50.28%	11.90%	4.13%	3.74%	3.96%	2.60%	6.06%	8.18%
226	TRA-1-60	0.15%	0.07%	0.24%	0.10%	0.18%	0.16%	0.38%	0.29%
227	TRA-1-81	1.25%	1.97%	0.34%	0.31%	0.29%	0.40%	0.92%	1.15%
228	Vß23	1.05%	1.53%	0.26%	0.16%	0.14%	0.16%	0.55%	0.65%
229	Vß8	0.23%	0.11%	0.35%	0.34%	0.32%	0.45%	1.78%	2.98%
230	CD326	0.69%	0.50%	0.39%	0.53%	0.32%	0.39%	1.10%	1.78%
231	mIgM	0.85%	0.91%	0.24%	0.11%	0.22%	0.20%	0.88%	1.13%
232	mIgG1	0.14%	0.03%	0.26%	0.23%	0.26%	0.30%	0.57%	0.77%
233	mIgG2a	1.00%	1.09%	0.71%	0.73%	0.81%	1.20%	2.83%	4.46%
234	mIgG2b	0.15%	0.05%	0.50%	0.67%	0.40%	0.48%	2.85%	4.86%
235	mIgG3	0.13%	0.02%	0.21%	0.12%	0.15%	0.18%	0.40%	0.54%
236	CD49f	23.02%	7.29%	28.35%	15.37%	3.12%	2.62%	94.69%	3.01%
237	CD104	0.18%	0.13%	0.27%	0.16%	0.18%	0.27%	1.07%	1.45%
238	CD120b	0.19%	0.09%	0.32%	0.19%	0.34%	0.38%	1.25%	1.84%
239	CD132	0.10%	0.06%	0.16%	0.14%	0.08%	0.09%	0.12%	0.16%
240	CD201	88.78%	8.59%	24.25%	21.03%	40.50%	36.48%	12.82%	18.48%
241	CD210	0.25%	0.11%	0.52%	0.54%	0.39%	0.57%	2.08%	2.96%
242	CD212	0.25%	0.06%	0.23%	0.10%	0.32%	0.39%	1.76%	2.68%
243	CD267	0.30%	0.16%	0.16%	0.14%	0.05%	0.04%	0.19%	0.18%
244	CD294	0.23%	0.05%	0.19%	0.17%	0.06%	0.05%	0.29%	0.31%
245	SSEA-3	0.20%	0.12%	0.29%	0.24%	0.33%	0.40%	1.14%	1.46%
246	Cutaneous Lymph Ark	0.29%	0.20%	0.26%	0.15%	0.40%	0.54%	0.80%	0.87%
247	Integrin ß7	0.25%	0.20%	0.51%	0.61%	0.51%	0.79%	1.71%	2.69%
248	rIgM	0.26%	0.21%	0.32%	0.18%	0.36%	0.55%	0.97%	1.20%
249	rIgG1	0.16%	0.10%	0.34%	0.27%	0.34%	0.49%	0.97%	1.10%
250	rIgG2a	0.14%	0.05%	0.43%	0.35%	0.29%	0.39%	1.34%	2.05%
251	rIgG2b	0.18%	0.14%	0.34%	0.26%	0.23%	0.39%	0.45%	0.41%

Table S2. The surface marker expression profile of TMSCs

		Un-differentiation		Adipogenesis					
				Total cells		Lipid-poor cells		Lipid-rich cells	
No.	Surface antigen	% positive	SD	% positive	SD	% positive	SD	% positive	SD
1	CD1a	0.24%	0.16%	0.26%	0.21%	0.10%	0.02%	0.25%	0.43%
2	CD1b	0.50%	0.54%	0.26%	0.12%	0.05%	0.04%	0.58%	0.55%
3	CD1d	0.29%	0.13%	0.20%	0.15%	0.11%	0.13%	0.26%	0.46%
4	CD2	0.27%	0.37%	0.14%	0.04%	0.03%	0.03%	0.58%	1.00%
5	CD3	0.79%	1.07%	0.24%	0.21%	0.07%	0.07%	0.26%	0.46%
6	CD4	0.26%	0.23%	0.18%	0.15%	0.06%	0.01%	0.00%	0.00%
7	CD4v4	1.09%	0.60%	0.59%	0.69%	0.17%	0.14%	1.11%	1.92%
8	CD5	0.36%	0.35%	0.22%	0.12%	0.07%	0.06%	0.70%	1.22%
9	CD6	0.32%	0.12%	0.14%	0.10%	0.03%	0.04%	0.43%	0.38%
10	CD7	0.32%	0.23%	0.15%	0.15%	0.07%	0.08%	0.00%	0.00%
11	CD8a	0.19%	0.17%	0.13%	0.12%	0.08%	0.06%	0.23%	0.40%
12	CD8b	0.37%	0.35%	0.30%	0.19%	0.12%	0.06%	1.22%	1.11%
13	CD9	99.60%	0.01%	84.00%	14.31%	90.97%	9.23%	70.36%	16.45%
14	CD10	99.79%	0.20%	95.23%	4.38%	98.29%	1.70%	92.86%	11.41%
15	CD11a	0.33%	0.37%	0.18%	0.09%	0.09%	0.05%	0.00%	0.00%
16	CD11b	0.94%	1.07%	0.19%	0.17%	0.09%	0.01%	0.25%	0.44%
17	CD11c	0.37%	0.42%	0.19%	0.07%	0.09%	0.06%	0.17%	0.30%
18	CD13	94.08%	8.07%	99.52%	0.46%	99.97%	0.04%	94.05%	4.75%
19	CD14	0.85%	0.60%	0.28%	0.18%	0.12%	0.09%	0.28%	0.48%
20	CD15	0.20%	0.12%	1.14%	0.78%	0.92%	0.72%	2.95%	2.18%
21	CD15s	0.26%	0.20%	0.21%	0.11%	0.09%	0.02%	0.36%	0.62%
22	CD16	0.17%	0.13%	0.17%	0.14%	0.02%	0.02%	0.19%	0.33%
23	CD18	0.19%	0.14%	0.10%	0.02%	0.08%	0.03%	0.00%	0.00%
24	CD19	0.21%	0.15%	0.17%	0.02%	0.06%	0.02%	0.29%	0.50%
25	CD20	0.52%	0.42%	0.32%	0.22%	0.11%	0.08%	0.78%	0.70%
26	CD21	0.18%	0.05%	0.17%	0.06%	0.04%	0.01%	0.19%	0.33%
27	CD22	0.27%	0.29%	0.13%	0.08%	0.07%	0.05%	0.21%	0.36%
28	CD23	0.20%	0.21%	0.15%	0.10%	0.06%	0.04%	0.68%	0.65%
29	CD24	0.59%	0.74%	0.11%	0.06%	0.12%	0.03%	0.21%	0.36%
30	CD25	0.23%	0.06%	0.17%	0.10%	0.07%	0.08%	0.55%	0.95%
31	CD26	65.88%	8.71%	16.07%	2.51%	16.79%	2.69%	24.56%	5.07%
32	CD27	0.39%	0.32%	0.41%	0.47%	0.14%	0.13%	0.89%	1.00%

33	CD28	0.22%	0.12%	0.19%	0.11%	0.08%	0.07%	0.23%	0.40%
34	CD29	97.36%	2.69%	62.32%	30.15%	76.08%	20.88%	87.61%	15.32%
35	CD30	0.10%	0.09%	0.15%	0.07%	0.07%	0.04%	0.69%	0.14%
36	CD31	0.18%	0.15%	0.12%	0.09%	0.02%	0.02%	0.50%	0.53%
37	CD32	0.24%	0.18%	0.23%	0.14%	0.08%	0.05%	0.83%	1.44%
38	CD33	0.26%	0.15%	0.21%	0.06%	0.07%	0.02%	0.58%	0.65%
39	CD34	0.94%	0.87%	2.59%	2.88%	1.82%	2.26%	8.73%	4.32%
40	CD35	0.13%	0.17%	0.15%	0.09%	0.08%	0.05%	0.74%	0.71%
41	CD36	5.72%	3.63%	3.43%	2.93%	1.39%	1.15%	36.28%	9.56%
42	CD37	0.27%	0.28%	0.19%	0.15%	0.07%	0.04%	0.63%	1.10%
43	CD38	0.25%	0.18%	0.87%	1.15%	1.03%	1.33%	1.00%	0.90%
44	CD39	8.14%	5.13%	39.52%	14.80%	44.08%	13.79%	28.47%	15.67%
45	CD40	16.68%	6.10%	15.24%	20.45%	19.95%	21.59%	16.68%	20.15%
46	CD41a	0.17%	0.15%	0.21%	0.12%	0.05%	0.03%	0.99%	0.69%
47	CD41b	0.17%	0.13%	0.13%	0.02%	0.05%	0.04%	0.67%	1.17%
48	CD42a	3.74%	2.18%	0.35%	0.35%	0.08%	0.08%	0.61%	0.64%
49	CD42b	0.23%	0.13%	0.17%	0.13%	0.03%	0.04%	0.51%	0.89%
50	CD43	0.17%	0.01%	0.15%	0.08%	0.03%	0.01%	0.50%	0.20%
51	CD44	99.89%	0.01%	99.64%	0.43%	99.92%	0.02%	94.51%	9.12%
52	CD45	0.16%	0.07%	0.70%	0.38%	2.38%	2.03%	0.94%	0.98%
53	CD45RA	1.03%	1.22%	0.19%	0.09%	0.09%	0.07%	0.44%	0.38%
54	CD45RB	0.11%	0.01%	0.13%	0.08%	0.08%	0.04%	0.22%	0.38%
55	CD45RO	0.62%	0.53%	0.39%	0.34%	0.17%	0.11%	1.08%	1.88%
56	CD46	99.78%	0.20%	95.45%	4.40%	98.64%	1.43%	87.02%	16.52%
57	CD47	99.76%	0.24%	89.69%	9.97%	98.08%	1.67%	72.03%	34.83%
58	CD48	0.23%	0.24%	0.40%	0.37%	0.12%	0.05%	2.37%	2.24%
59	CD49a	92.36%	5.33%	72.91%	26.25%	81.17%	19.43%	77.58%	14.43%
60	CD49b	99.57%	0.20%	86.16%	10.86%	93.70%	4.89%	79.84%	14.55%
61	CD49c	99.48%	0.32%	38.07%	24.68%	40.49%	25.93%	63.66%	18.35%
62	CD49d	82.45%	1.29%	5.19%	5.69%	5.57%	6.01%	5.68%	3.37%
63	CD49e	99.67%	0.21%	89.01%	10.53%	96.35%	4.29%	84.43%	19.83%
64	CD50	0.31%	0.21%	0.18%	0.08%	0.11%	0.08%	0.91%	0.48%
65	CD51/61	67.91%	6.85%	10.88%	13.02%	12.29%	15.93%	12.63%	13.42%
66	CD53	0.16%	0.17%	0.12%	0.07%	0.07%	0.04%	0.53%	0.54%
67	CD54	92.63%	2.96%	8.10%	5.20%	9.19%	5.16%	12.94%	4.12%
68	CD55	99.84%	0.15%	85.76%	11.81%	92.86%	5.85%	80.07%	16.97%
69	CD56	1.15%	0.73%	0.50%	0.34%	0.50%	0.41%	1.63%	0.73%
70	CD57	11.25%	6.07%	3.27%	2.17%	2.96%	2.16%	6.95%	1.75%

71	CD58	99.04%	1.02%	56.58%	35.42%	71.37%	26.26%	64.19%	29.98%
72	CD59	99.97%	0.03%	98.86%	0.97%	99.95%	0.03%	94.48%	5.80%
73	CD61	61.16%	3.32%	7.77%	9.77%	8.65%	10.75%	14.21%	8.49%
74	CD62E	0.11%	0.09%	0.18%	0.02%	0.05%	0.05%	0.60%	0.55%
75	CD62L	0.21%	0.02%	0.08%	0.05%	0.03%	0.03%	0.00%	0.00%
76	CD62P	0.21%	0.08%	0.19%	0.12%	0.06%	0.04%	0.74%	1.29%
77	CD63	99.36%	0.62%	87.25%	10.76%	93.52%	6.40%	91.57%	7.37%
78	CD64	0.13%	0.06%	0.17%	0.07%	0.05%	0.03%	0.74%	0.13%
79	CD66(a,c,d,e)	0.35%	0.26%	0.31%	0.21%	0.09%	0.06%	0.33%	0.31%
80	CD66b	0.57%	0.27%	0.23%	0.18%	0.10%	0.06%	0.21%	0.37%
81	CD66f	0.15%	0.01%	0.23%	0.11%	0.11%	0.08%	0.63%	0.67%
82	CD69	0.14%	0.11%	0.13%	0.05%	0.07%	0.06%	0.23%	0.40%
83	CD70	0.39%	0.26%	0.18%	0.05%	0.04%	0.03%	1.00%	1.21%
84	CD71	98.22%	1.62%	43.68%	30.24%	51.48%	28.13%	77.08%	23.41%
85	CD72	0.34%	0.32%	0.23%	0.13%	0.13%	0.12%	0.47%	0.40%
86	CD73	99.89%	0.07%	98.32%	1.51%	99.54%	0.38%	94.37%	5.32%
87	CD74	0.20%	0.06%	0.38%	0.21%	0.22%	0.13%	0.80%	0.84%
88	CD75	2.48%	2.21%	0.32%	0.26%	0.16%	0.10%	0.77%	1.33%
89	CD77	4.85%	2.17%	3.03%	3.80%	2.45%	3.06%	5.56%	8.83%
90	CD79b	4.87%	5.06%	1.14%	1.51%	0.24%	0.27%	4.28%	5.32%
91	CD80	0.17%	0.02%	0.20%	0.17%	0.05%	0.02%	1.01%	0.93%
92	CD81	99.67%	0.25%	98.76%	1.12%	99.87%	0.10%	89.14%	8.25%
93	CD83	0.43%	0.52%	0.28%	0.08%	0.16%	0.10%	0.89%	0.27%
94	CD84	0.14%	0.11%	0.22%	0.17%	0.10%	0.02%	0.97%	0.95%
95	CD85	0.28%	0.18%	0.13%	0.06%	0.07%	0.06%	0.48%	0.44%
96	CD86	0.20%	0.17%	0.18%	0.09%	0.04%	0.02%	0.52%	0.47%
97	CD87	0.11%	0.04%	0.17%	0.05%	0.03%	0.02%	0.23%	0.39%
98	CD88	0.10%	0.05%	0.14%	0.11%	0.05%	0.06%	0.23%	0.40%
99	CD89	0.12%	0.01%	0.18%	0.09%	0.10%	0.08%	0.18%	0.31%
100	CD90	99.54%	0.40%	96.93%	2.86%	98.91%	1.17%	93.82%	8.35%
101	CD91	33.51%	25.96%	3.92%	0.87%	3.61%	0.53%	11.08%	4.84%
102	CDw93	0.10%	0.09%	0.22%	0.10%	0.04%	0.03%	1.20%	1.50%
103	CD94	0.12%	0.07%	0.12%	0.05%	0.06%	0.01%	0.33%	0.57%
104	CD95	98.56%	1.16%	84.42%	13.46%	94.83%	4.52%	61.57%	16.35%
105	CD97	29.54%	32.34%	1.59%	1.97%	2.56%	3.40%	1.69%	1.47%
106	CD98	97.37%	2.41%	76.83%	28.57%	86.05%	20.89%	81.49%	25.78%
107	CD99	99.69%	0.24%	85.48%	12.57%	94.04%	5.89%	73.87%	22.15%
108	CD99R	90.84%	6.82%	27.83%	43.68%	31.33%	46.31%	22.93%	32.06%

109	CD100	0.10%	0.08%	0.17%	0.16%	0.04%	0.04%	0.25%	0.44%
110	CD102	4.86%	0.49%	0.59%	0.69%	0.44%	0.61%	0.76%	0.74%
111	CD103	0.11%	0.05%	0.09%	0.07%	0.04%	0.02%	0.46%	0.40%
112	CD105	94.28%	3.67%	61.69%	31.09%	77.15%	18.90%	55.06%	20.87%
113	CD106	27.95%	9.69%	8.19%	3.64%	12.07%	3.36%	4.17%	0.90%
114	CD107a	17.42%	8.89%	2.18%	1.10%	1.31%	0.58%	4.58%	2.18%
115	CD107b	4.68%	3.27%	1.97%	0.81%	0.80%	0.34%	7.60%	4.45%
116	CD108	32.87%	21.38%	0.98%	1.00%	0.67%	0.64%	2.51%	1.84%
117	CD109	1.55%	1.75%	0.23%	0.10%	0.13%	0.03%	0.92%	0.45%
118	CD112	1.81%	2.09%	0.33%	0.23%	0.05%	0.07%	0.45%	0.41%
119	CD114	0.11%	0.03%	0.14%	0.11%	0.05%	0.03%	0.69%	0.67%
120	CD116	0.35%	0.22%	1.15%	1.88%	0.88%	1.47%	0.99%	1.71%
121	CD117	0.05%	0.06%	0.16%	0.08%	0.09%	0.03%	1.17%	0.28%
122	CD118	0.06%	0.02%	0.13%	0.07%	0.06%	0.01%	0.35%	0.32%
123	CD119	2.36%	0.52%	6.58%	9.70%	8.63%	11.59%	7.42%	8.70%
124	CD120a	2.66%	2.45%	0.39%	0.54%	0.16%	0.13%	0.00%	0.00%
125	CD121a	8.19%	1.94%	14.00%	17.69%	18.73%	20.76%	10.44%	10.94%
126	CD121b	0.13%	0.10%	0.17%	0.11%	0.07%	0.05%	0.48%	0.83%
127	CD122	0.13%	0.04%	0.12%	0.13%	0.09%	0.06%	0.00%	0.00%
128	CD123	0.29%	0.30%	0.11%	0.12%	0.04%	0.01%	0.68%	0.96%
129	CD124	0.19%	0.08%	0.14%	0.11%	0.09%	0.04%	1.21%	1.71%
130	CD126	18.66%	15.83%	0.17%	0.12%	0.10%	0.05%	0.31%	0.43%
131	CD127	0.26%	0.21%	0.16%	0.11%	0.05%	0.03%	0.22%	0.38%
132	CD128b(CD182)	5.07%	4.53%	0.11%	0.06%	0.09%	0.06%	0.21%	0.37%
133	CD130	50.29%	5.46%	2.86%	4.59%	3.11%	5.10%	3.38%	5.85%
134	CD134	0.08%	0.04%	0.17%	0.08%	0.06%	0.07%	0.64%	0.57%
135	CD135	0.13%	0.06%	0.16%	0.09%	0.07%	0.01%	0.00%	0.00%
136	CD137	0.14%	0.07%	0.16%	0.10%	0.10%	0.06%	0.70%	1.21%
137	CD137 Ligand	0.12%	0.13%	0.18%	0.16%	0.06%	0.02%	0.80%	0.70%
138	CD138	0.18%	0.18%	0.14%	0.05%	0.07%	0.00%	0.41%	0.36%
139	CD140a	92.04%	2.99%	80.45%	10.23%	90.56%	5.45%	50.36%	8.93%
140	CD140b	98.35%	0.78%	97.54%	2.39%	99.38%	0.53%	86.54%	10.82%
141	CD141	11.73%	2.74%	6.70%	7.59%	7.04%	7.86%	4.73%	4.25%
142	CD142	85.21%	1.94%	60.81%	27.23%	67.86%	22.73%	75.18%	18.95%
143	CD144	0.09%	0.03%	0.16%	0.05%	0.04%	0.04%	0.18%	0.31%
144	CD146	6.95%	2.77%	7.18%	2.82%	4.34%	2.17%	84.02%	11.25%
145	CD147	99.81%	0.05%	99.35%	0.50%	99.88%	0.10%	95.35%	4.92%
146	CD150	0.17%	0.16%	0.16%	0.08%	0.05%	0.05%	0.42%	0.72%

147	CD151	99.23%	0.22%	68.71%	28.01%	81.88%	16.59%	77.81%	22.26%
148	CD152	1.16%	0.65%	0.29%	0.22%	0.10%	0.05%	0.78%	0.94%
149	CD153	0.62%	0.71%	0.99%	1.38%	0.32%	0.22%	4.62%	8.01%
150	CD154	0.09%	0.03%	0.16%	0.09%	0.04%	0.04%	0.00%	0.00%
151	CD158a	0.13%	0.06%	0.18%	0.16%	0.06%	0.00%	0.80%	1.12%
152	CD158b	0.22%	0.08%	0.15%	0.04%	0.08%	0.02%	0.32%	0.45%
153	CD161	0.18%	0.06%	0.10%	0.08%	0.07%	0.01%	0.78%	1.10%
154	CD162	2.26%	0.40%	0.43%	0.44%	0.08%	0.05%	5.33%	4.64%
155	CD163	0.10%	0.06%	0.10%	0.08%	0.03%	0.04%	0.19%	0.33%
156	CD164	99.48%	0.46%	60.24%	27.27%	68.52%	22.44%	63.19%	17.88%
157	CD165	92.98%	4.72%	31.58%	46.49%	37.10%	48.59%	26.39%	31.13%
158	CD166	98.53%	1.18%	66.20%	24.78%	78.55%	16.33%	59.53%	11.00%
159	CD171	0.32%	0.21%	0.15%	0.06%	0.05%	0.04%	0.45%	0.46%
160	CD172b	0.07%	0.01%	0.16%	0.08%	0.05%	0.05%	0.53%	0.61%
161	CD177	0.27%	0.12%	0.12%	0.05%	0.06%	0.03%	0.55%	0.55%
162	CD178	0.13%	0.09%	0.12%	0.07%	0.06%	0.03%	0.25%	0.43%
163	CD180	0.15%	0.08%	0.21%	0.16%	0.06%	0.05%	0.59%	0.30%
164	CD181	4.14%	3.80%	0.40%	0.42%	0.11%	0.15%	0.49%	0.69%
165	CD183	4.32%	3.66%	0.68%	0.83%	0.20%	0.25%	2.04%	1.44%
166	CD184	0.34%	0.33%	0.11%	0.08%	0.01%	0.01%	0.59%	0.01%
167	CD193	1.52%	1.22%	0.33%	0.34%	0.09%	0.03%	1.26%	1.49%
168	CD195	0.71%	0.04%	0.31%	0.37%	0.13%	0.18%	0.79%	0.80%
169	CD196	1.95%	0.71%	0.15%	0.10%	0.05%	0.04%	0.56%	0.49%
170	CD197	1.19%	0.28%	0.90%	1.32%	1.10%	1.78%	0.38%	0.37%
171	CD200	0.55%	0.35%	0.77%	1.04%	0.61%	0.81%	4.34%	7.07%
172	CD205	1.30%	1.01%	0.46%	0.28%	0.52%	0.59%	0.53%	0.18%
173	CD206	0.15%	0.01%	0.15%	0.05%	0.05%	0.05%	0.17%	0.29%
174	CD209	3.59%	1.40%	1.25%	1.20%	0.40%	0.22%	7.15%	9.81%
175	CD220	0.22%	0.13%	0.13%	0.13%	0.06%	0.03%	0.42%	0.73%
176	CD221	15.19%	2.21%	1.34%	1.87%	1.09%	1.67%	0.54%	0.60%
177	CD226	0.11%	0.03%	0.13%	0.09%	0.06%	0.05%	0.00%	0.00%
178	CD227	36.44%	16.39%	6.82%	10.77%	7.60%	12.16%	10.22%	16.35%
179	CD229	0.20%	0.05%	0.13%	0.16%	0.06%	0.03%	0.00%	0.00%
180	CD231	0.32%	0.26%	0.11%	0.08%	0.04%	0.01%	0.23%	0.39%
181	CD235a	0.23%	0.03%	0.14%	0.13%	0.08%	0.06%	0.39%	0.68%
182	CD243	3.47%	3.95%	0.28%	0.25%	0.09%	0.04%	0.92%	0.42%
183	CD244	0.19%	0.01%	0.12%	0.05%	0.04%	0.02%	0.47%	0.51%
184	CD255	0.16%	0.04%	0.15%	0.15%	0.08%	0.08%	0.83%	0.80%

185	CD268	0.14%	0.02%	0.13%	0.05%	0.05%	0.03%	0.93%	0.98%
186	CD271	0.62%	0.17%	2.00%	1.91%	2.14%	1.87%	2.25%	2.56%
187	CD273	55.40%	9.32%	2.02%	1.68%	1.39%	1.27%	4.74%	5.30%
188	CD274	29.90%	10.87%	0.34%	0.32%	0.19%	0.17%	0.47%	0.81%
189	CD275	1.08%	1.09%	3.93%	4.23%	5.42%	5.14%	4.28%	2.60%
190	CD278	0.15%	0.00%	0.15%	0.11%	0.04%	0.02%	0.35%	0.31%
191	CD279	0.13%	0.02%	0.21%	0.07%	0.04%	0.04%	0.51%	0.45%
192	CD282	0.14%	0.10%	0.23%	0.14%	0.03%	0.02%	2.03%	2.63%
193	CD305	0.17%	0.06%	0.88%	0.36%	0.09%	0.07%	20.87%	14.68%
194	CD309	0.73%	0.64%	0.14%	0.07%	0.05%	0.01%	0.00%	0.00%
195	CD314	0.11%	0.02%	0.23%	0.23%	0.07%	0.07%	0.00%	0.00%
196	CD321	0.51%	0.38%	1.10%	0.56%	0.27%	0.21%	16.64%	18.06%
197	CDw327	0.21%	0.07%	0.11%	0.11%	0.04%	0.04%	0.71%	0.75%
198	CDw328	0.11%	0.04%	0.20%	0.12%	0.10%	0.01%	0.45%	0.39%
199	CD329	0.20%	0.11%	0.21%	0.16%	0.09%	0.02%	0.28%	0.48%
200	CD335	0.20%	0.11%	0.17%	0.05%	0.09%	0.03%	0.72%	0.72%
201	CD336	0.21%	0.16%	0.23%	0.13%	0.12%	0.04%	0.19%	0.32%
202	CD337	0.48%	0.26%	1.92%	0.99%	0.51%	0.35%	3.32%	4.55%
203	CD338	0.16%	0.04%	0.32%	0.19%	0.13%	0.01%	0.33%	0.57%
204	CD340	69.14%	8.69%	24.60%	37.09%	34.34%	41.23%	13.76%	18.63%
205	abTCR	0.83%	0.65%	0.30%	0.31%	0.17%	0.14%	0.31%	0.54%
206	b2microglobulin	96.74%	2.72%	96.77%	3.24%	99.37%	0.68%	82.24%	19.24%
207	BLTR-1	0.14%	0.08%	0.18%	0.05%	0.09%	0.03%	0.58%	1.00%
208	CLIP	0.14%	0.05%	0.14%	0.14%	0.10%	0.04%	0.00%	0.00%
209	CMRF-44	0.43%	0.45%	0.15%	0.14%	0.05%	0.01%	0.00%	0.00%
210	CDRF-56	0.67%	0.70%	0.21%	0.05%	0.15%	0.09%	0.37%	0.65%
211	EGF-R	94.91%	1.41%	25.36%	13.16%	39.90%	16.13%	15.44%	12.69%
212	fMLP-R	0.35%	0.13%	0.53%	0.75%	0.61%	0.93%	0.21%	0.36%
213	gdTCR	0.14%	0.03%	0.38%	0.46%	0.41%	0.52%	0.33%	0.57%
214	Hem.progenitor cell	47.35%	2.07%	20.66%	29.43%	24.37%	31.00%	19.16%	29.82%
215	HLA-A,B,C	99.24%	0.15%	98.08%	1.89%	99.78%	0.24%	83.00%	18.56%
216	HLA-A2	0.14%	0.08%	0.71%	0.99%	0.82%	1.33%	0.91%	1.57%
217	HLA-DQ	7.33%	9.87%	0.21%	0.05%	0.14%	0.12%	0.45%	0.39%
218	HLA-DR	0.49%	0.03%	0.13%	0.02%	0.09%	0.03%	0.22%	0.38%
219	HLA-DR,DP,DQ	0.22%	0.02%	0.24%	0.27%	0.09%	0.06%	0.00%	0.00%
220	Invariant NKT	0.12%	0.03%	1.07%	1.57%	1.10%	1.81%	0.98%	1.24%

221	Disialoganglioside GD2	20.45%	1.71%	26.10%	14.43%	29.42%	14.73%	23.72%	18.70%
222	MIC A/B	3.59%	1.66%	0.26%	0.06%	0.13%	0.06%	0.58%	0.53%
223	NKB1	0.13%	0.03%	0.11%	0.03%	0.04%	0.02%	0.91%	0.80%
224	SSEA-1	0.22%	0.06%	1.05%	0.92%	1.01%	0.87%	1.77%	1.55%
225	SSEA-4	37.86%	1.87%	9.05%	12.73%	9.67%	12.88%	13.80%	21.60%
226	TRA-1-60	0.16%	0.04%	0.17%	0.07%	0.08%	0.06%	0.55%	0.62%
227	TRA-1-81	0.12%	0.07%	0.14%	0.04%	0.06%	0.04%	1.11%	1.11%
228	Vß23	0.25%	0.08%	0.13%	0.09%	0.14%	0.07%	0.43%	0.37%
229	Vß8	0.15%	0.05%	0.24%	0.19%	0.08%	0.02%	0.96%	1.12%
230	CD326	0.52%	0.07%	0.42%	0.41%	0.14%	0.12%	0.41%	0.72%
231	mIgM	0.64%	0.49%	0.34%	0.35%	0.13%	0.06%	0.20%	0.35%
232	mIgG1	0.09%	0.00%	0.16%	0.09%	0.06%	0.02%	0.78%	1.36%
233	mIgG2a	1.38%	0.66%	0.95%	1.45%	0.27%	0.36%	3.95%	6.39%
234	mIgG2b	0.22%	0.06%	0.19%	0.17%	0.07%	0.02%	0.41%	0.36%
235	mIgG3	0.33%	0.09%	0.13%	0.17%	0.08%	0.04%	0.72%	0.77%
236	CD49f	41.88%	5.35%	4.62%	5.35%	3.77%	4.76%	25.70%	21.29%
237	CD104	0.22%	0.15%	0.14%	0.11%	0.12%	0.12%	0.22%	0.38%
238	CD120b	1.00%	0.29%	1.52%	2.21%	1.82%	2.64%	1.57%	2.19%
239	CD132	0.17%	0.09%	0.41%	0.48%	0.40%	0.53%	0.39%	0.68%
240	CD201	46.07%	12.37%	18.74%	18.27%	23.46%	18.73%	19.31%	20.19%
241	CD210	0.21%	0.02%	0.18%	0.06%	0.09%	0.02%	0.61%	0.59%
242	CD212	0.17%	0.11%	0.16%	0.09%	0.06%	0.04%	0.97%	0.85%
243	CD267	0.17%	0.08%	0.14%	0.13%	0.05%	0.04%	0.59%	0.52%
244	CD294	0.17%	0.16%	0.26%	0.21%	0.13%	0.09%	0.72%	1.25%
245	SSEA-3	0.20%	0.04%	0.21%	0.10%	0.08%	0.03%	0.45%	0.39%
246	Cutaneous Lymph Ark	0.17%	0.06%	0.12%	0.04%	0.10%	0.03%	0.44%	0.39%
247	Integrin ß7	0.13%	0.01%	0.16%	0.10%	0.10%	0.04%	0.70%	0.65%
248	rIgM	0.23%	0.11%	0.17%	0.03%	0.10%	0.05%	0.56%	0.61%
249	rIgG1	0.18%	0.08%	0.14%	0.03%	0.08%	0.02%	0.00%	0.00%
250	rIgG2a	0.15%	0.11%	0.13%	0.03%	0.07%	0.07%	0.59%	1.02%
251	rIgG2b	0.15%	0.04%	0.12%	0.05%	0.05%	0.02%	0.45%	0.79%

Table S3. Donor's information

	Donor	Gender/Age	Cat. Number	Lot Number
BMSCs	1	M/62	C-12974	413Z021
	2	M/63	C-12974	465Z016
TMSCs	1	M/5		ETMSC-15-001
	2	M/5		ETMSC-17-039
	3	M/7		ETMSC-18-006
	4	M/13		ETMSC-17-040
	5	M/16		ETMSC-17-041
